# Development of a prognostic model for anoikis and identifies hub genes in hepatocellular carcinoma

**DOI:** 10.1038/s41598-023-41139-9

**Published:** 2023-09-07

**Authors:** Zhiwei Zhong, Fuchun Xie, Jiajun Yin, Hua Zhao, Yuehan Zhou, Kun Guo, Rongkuan Li, Qimin Wang, Bo Tang

**Affiliations:** 1 Department of Infectious Disease, The Second Affiliated Hospital of Dalian Medical University, Dalian, 116023 People’s Republic of China; 2 Department of Radiology, Guangdong Provincial People’s Hospital, Guangdong Academy of Medical Sciences, Guangzhou, 510080 People’s Republic of China; 3 Department of General Surgery, Affiliated Zhongshan Hospital of Dalian University, Dalian, 116300 People’s Republic of China; 4 Department of Hematology, The Second Affiliated Hospital of Dalian Medical University, Dalian, 116023 Liaoning People’s Republic of China; 5 Department of Pathology, The Second Affiliated Hospital of Dalian Medical University, Dalian, 116023 Liaoning People’s Republic of China

**Keywords:** Cancer, Prognostic markers

## Abstract

Considering the high fatality of hepatocellular carcinoma (HCC), current prognostic systems are insufficient to accurately forecast HCC patients' outcomes. In our study, nine anoikis‑related genes (PTRH2, ITGAV, ANXA5, BIRC5, BDNF, BSG, DAP3, SKP2, and EGF) were determined to establish a risk scoring model using LASSO regression, which could be validated in ICGC dataset. Kaplan–Meier curves and time-dependent receiver operating characteristic (ROC) curve analysis confirmed the risk score possessed an accurate predictive value for the prognosis of HCC patients. The high-risk group showed a higher infiltration of aDCs, macrophages, T-follicular helper cells, and Th2 cells. Besides, PD-L1 was significantly higher in the high-risk group compared to the low-risk group. Several anoikis‑related genes, such as ANX5, ITGAV, BDNF and SKP2, were associated with drug sensitivity in HCC. Finally, we identified BIRC5 and SKP2 as hub genes among the nine model genes using WGCNA analysis. BIRC5 and SKP2 were over-expressed in HCC tissues, and their over-expression was associated with poor prognosis, no matter in our cohort by immunohistochemical staining or in the TCGA cohort by mRNA-Seq. In our cohort, BIRC5 expression was highly associated with the T stage, pathologic stage, histologic grade and AFP of HCC patients. In general, our anoikis-related risk model can enhance the ability to predict the survival outcomes of HCC patients and provide a feasible therapeutic strategy for immunotherapy and drug resistance in HCC. BIRC5 and SKP2 are hub genes of anoikis‑related genes in HCC.

## Introduction

Hepatocellular carcinoma (HCC) remains a great challenge in terms of global health, for it is the sixth most common type of cancer and the third leading cause of cancer-related mortality worldwide. Studies have shown that about 906,000 new cases (4.7%) and 830,000 deaths (8.3%) worldwide in 2020^1^. At present, there are multiple therapeutic strategies for HCC, including surgical resection, liver transplantation, radiofrequency ablation, vascular catheterization and chemotherapy^2,3^. However, the prognosis of HCC remains poor, with a 5-year survival rate of 20%^4^. Therefore, early prediction for prognosis is of great significance to improve the survival outcomes of HCC patients. Considering that cirrhosis is the basis of HCC in most patients, the prognosis of HCC depends on the tumor burden, the degree of liver dysfunction, and the patient's performance status^5^. Several prognostic systems have been proposed for patients with HCC in the last three decades, including the BCLC system, CNLC classification, the MESH score, and the CLIP score^6^. Nevertheless, it is rather difficult to confirm the best prognostic system that could be widely used for all HCC patients in clinical practices^7^. As a result, current prognostic systems are not sufficient to accurately forecast the outcomes of HCC patients. In addition, the identification of molecular biomarkers associated with HCC prognosis attracted considerable attention, but the number of prognostic model is limited. Hence, it is necessary to develop a new tool based on prognostic biomarkers to achieve an optimal evaluation of survival outcomes in HCC patients.

Anoikis is a type of programmed apoptosis initiated by detachment of cells from the extracellular matrix (ECM). It is a mechanism that contributes to tissue homeostasis by eliminating detached or misplaced cells under physiological or pathological conditions. Besides, anoikis can limit cancer progression by preventing the dissemination of cancer cells to distant organs^8^. Studies have shown that the induction of the anoikis occurs through the interaction of the intrinsic pathway (mitochondria approach) and extrinsic pathway (death receptor approach). It has been reported cancer cells develop anoikis resistance by escaping the death signals and activating the pro-survival signals^9,10^. Meanwhile, acquisition of anoikis resistance is a hallmark of cancer cell invasiveness, metastasis, treatment resistance, and relapse^11^. In addition, researchers have identified that different players, including cell adhesion molecules, growth factors, hypoxia, stemness, autophagy, long non-coding RNAs, and signaling pathways, are associated with anoikis resistance, allowing cell survival in the cellular environment^12–15^. However, the precise mechanisms involved in anoikis resistance are not fully understood. Therefore, the early investigation into the mechanisms of anoikis resistance may greatly help to improve the prognostic assessment of cancer and further efficacious treatments for cancer.

Currently, enormous efforts have shown that many anoikis-related genes are associated with the proliferation of several tumors, including gastric cancer, colorectal cancer, lung cancer, and pancreatic cancer^16–18^. For example, GDH1 provides signals of anoikis resistance and tumor proliferation in LKB1-deficient lung cancer. Also, GDH1 knockdown can not only improve cell sensitivity to anoikis induction but also reduce tumor metastasis^19^. In high-grade serous ovarian carcinoma, CBX2 has been verified to drive anoikis resistance and tumor metastasis^20^. TMPRSS4, a cell surface-anchored serine protease, is reported to promote anoikis resistance, enhancing survival of circulating tumor cells and leading to early metastasis in prostate cancer^21^. Therefore, anoikis‑related genes are considered as promising diagnostic markers and potential therapeutic targets. However, the construction of a prognostic risk model based on anoikis‑related genes remains unclear in HCC. Hence, we exploit the relationship between anoikis‑related genes and HCC clinical information to forecast the outcomes of HCC patients.

Given the limited effects of standard treatments, increasing attention is focused on immunotherapy in recent years^22,23^. Studies highlighted immune cells producing cytokines and components of the ECM as response to liver injury, which promotes the survival of cancer stem cells^24^. Currently, immunotherapy is considered as an additional treatment and witness substantial progress^25,26^. Thus, exploring the underlying mechanism between the immune system and cancer to enhance the immunotherapy of HCC is urgently required.

Herein, multiple mRNA expression databases were utilized to develop and validate a risk scoring model related to anoikis for HCC. Moreover, we explored the potential relevance between the prognostic risk score and tumor stemness, immunity as well as drug sensitivity. Finally, we identified BIRC5 and SKP2 as hub genes in HCC. They are over-expressed in HCC tissues, and were associated with poor prognosis, no matter in the TCGA cohort or our TMA cohort. All of these might not only promote the capability to forecasting the survival outcomes of HCC patients, but also provide prominent reference for molecular mechanisms of immunotherapy and drug resistance in HCC.

## Methods

### Data collection process

The mRNA expression matrix of 374 liver hepatocellular carcinoma samples and 50 adjacent normal samples, and the corresponding clinicopathological information were downloaded from The Cancer Genome Atlas (TCGA) database (https://portal.gdc.cancer.gov/). Besides, we also downloaded the mRNA expression data and the clinical information of another 231 HCC patients from the International Cancer Genome Consortium (ICGC) database (https://dcc.icgc.org/). From the GeneCards (https://www.genecards.org/), 82 anoikis‑related genes were extracted by screening with a relevance score > 1.8.

### Identification of prognostic anoikis‑related DEGs and functional analysis

To obtain differentially expressed genes (DEGs), we used the “limma” package of R language to conduct differential analysis on the expression of the above 82 anoikis‑related genes in normal and tumor samples from the TCGA database. Thirty-eight anoikis‑related DEGs were obtained, and the screening criteria were as follows: |log (fold change)| > 1 and adjusted *p* < 0.05^27^. Through univariate Cox regression analysis, 26 anoikis‑related DEGs were associated with the prognosis of HCC (prognostic anoikis‑related DEGs, *p* < 0.05).

STRING Database (Version 11.0, https://STRINGDb.org/) was used to map the protein–protein interaction (PPI) network of these 26 prognostic anoikis‑related DEGs. The Kyoto Encyclopedia of Genes and Genomes (KEGG) and Gene Ontology (GO) analyses were performed based on the aforementioned prognostic anoikis‑related DEGs^28^.

### Establishment and verification of anoikis risk scoring model

The least absolute contraction and selection operator (LASSO) method was used to punish the factor data. The risk score was calculated according to the standardized expression level of each gene and its corresponding regression coefficient. Then an anoikis risk scoring model was established with nine genes. According to the median risk score, all cases were divided into the high-risk scoring group and the low-risk scoring group. The “stats” and “Rtsne” packets of R language were used for principal component analysis (PCA) and t-distribution random neighbor embedding (t-SNE) to detect the internal correlation between the two groups. Kaplan–Meier (K-M) curve was used to evaluate the survival difference between the two groups. The reliability of the risk model was analyzed by using the “time-dependent receiver operating characteristic (ROC)” R package. The statistical significance of all analyses was set as *p* < 0.05. The prognostic accuracy of the anoikis risk score was validated in the ICGC cohort. Univariate and multivariate Cox analyses were used to analyze the independent prognostic role of age, gender, grade, stage, and the anoikis risk score in the TCGA cohort and the ICGC cohort.

### Associations of the anoikis risk scoring model and immune cells, immune-related functions, stemness score and PD-L1 expression

We used single sample gene set enrichment analysis (ssGSEA) via the R packages "GSVA" and "GSEABase" to evaluate the filtering scores of 16 immune cell types and the activity of 13 immune-related functions. The meaningful value is *p* < 0.05. The R packages "limma" and "ggpubr" were used to further visualize the relationship between each immune subtype and risk score. Spearman’s correlation test was used to test the correlation between the risk scores and tumor stemness, immune, stromal, and ESTIMATE scores. Pearson analysis was used to test the association of risk scores with the expression of the key immunomodulator PD-L1.

### Drug sensitivity analysis

The data of RNA-seq expression profiles and DTP NCI-60 compound activity data were downloaded from CellMiner (https://discover.nci.nih.gov/cellminer) to analyze the drug sensitivity of nine model genes in HCC. Then we chose drugs approved by FDA to further analyze the correlation between nine model genes expression and drug sensitivity through R package “impute”, “limma”, “ggplot2”, and “ggpubr”. Pearson correlation analysis was carried out to screen the first 16 gene drugs according to the Pearson correlation coefficient, to study the relationship between prognosis anoikis-related genes and drug sensitivity.

### Patients and tissue samples

This study was approved by the Human Ethics Committee of the Second Hospital of Dalian Medical University to collect tissue samples. We collected paraffin-embedded liver cancer tissues and adjacent non-tumor tissues from 88 HCC patients who underwent surgical treatment at the Second Hospital of Dalian Medical University from 2017 to 2019. These HCC patients ranged from 35 to 77 years old, including 19 females and 69 males. All patients signed an informed consent form as required by the ethics committee. Complete clinical data and follow-up information were available for all patients.

### Immunohistochemistry

We first produced tissue microarrays (TMAs) of these 88 HCC tissues and matched adjacent non-cancerous tissues. Three μm sections were obtained from paraffin-embedded tissues for BIRC5 and SKP2 immunohistochemical studies according to standard procedures. Briefly, a brief protein hydrolysis digestion was performed using IHC enzyme antigen recovery agent and peroxidase blocking was performed using 3% H_2_O_2_. Then sections were incubated overnight at 4 °C with BIRC5 (Survivin) polyclonal antibody (1:200, Bioss, China) or SKP2 polyclonal antibody (1:100, Bioss, China). After PBS wash to remove the unbound primary antibody, the secondary antibody (enzyme-labeled goat anti-rabbit IgG, dilution, 1:1000) was coupled with peroxidase at incubate for 30 min at 37 °C. Sections were stained with 3,3′diaminobenzidine (DAB) substrate and washed with water. Finally, sections were counterstained with hematoxylin for 20 s, then washed with hydrochloric acid and ammonia, dehydrated, cleared, and cover-stained.

### Evaluation of staining

Stained sections were scanned using an Aperio GT450 section scanner (Leica Biosystems Imaging, USA), and markers were pathologically evaluated in the Aperio ImageScope image viewer according to the percentage of positive cells and staining intensity. Protein expression was scored by two independent and experienced pathologists. The degree of staining was defined as the percentage of hepatocytes or paracancerous tissue in the positively stained area as a percentage of the entire tissue area, scored on a scale of 1–4, 1 (0–25%); 2 (26–50); 3 (51–75%); 4 (76–100%). Staining intensity was defined as a score of 0–3, 0 (negative); 1 (weakly positive); 2 (positive); 3 (strongly positive). The percentages and intensity scores were summed to give each patient a final immunoreactivity score (IRS). Low expression of BIRC5 was judged when the IRS was 1 and high expression was judged when the score was 2–3. Low expression of SKP2 was judged when the IRS was 1–3 and high expression was judged when the IRS was 4–6.

### Statistical analysis

SPSS 26.0 software was used for statistical analysis, and the chi-square test was used to analyze the relationship between protein/mRNA expression and clinicopathological characteristics of HCC patients. Univariate and multivariate Cox regression analyses were used to analyze the factors influencing survival in HCC patients. The Kaplan–Meier method was used for the detection of survival analysis and the log-rank test was used to assess the differences. The relative risk of death was expressed as an adjusted hazard ratio (HR) with a 95% confidence interval (CI). If *p* < 0.05 all tests were considered statistically significant.

### Institutional review board statement

The study was conducted in accordance with the Declaration of Helsinki, and The Ethics Committee of the Second Affiliated Hospital of Dalian Medical University approved this research.

## Results

### Screening and identification of prognostic anoikis‑related DEGs in HCC

**Figure 1:**
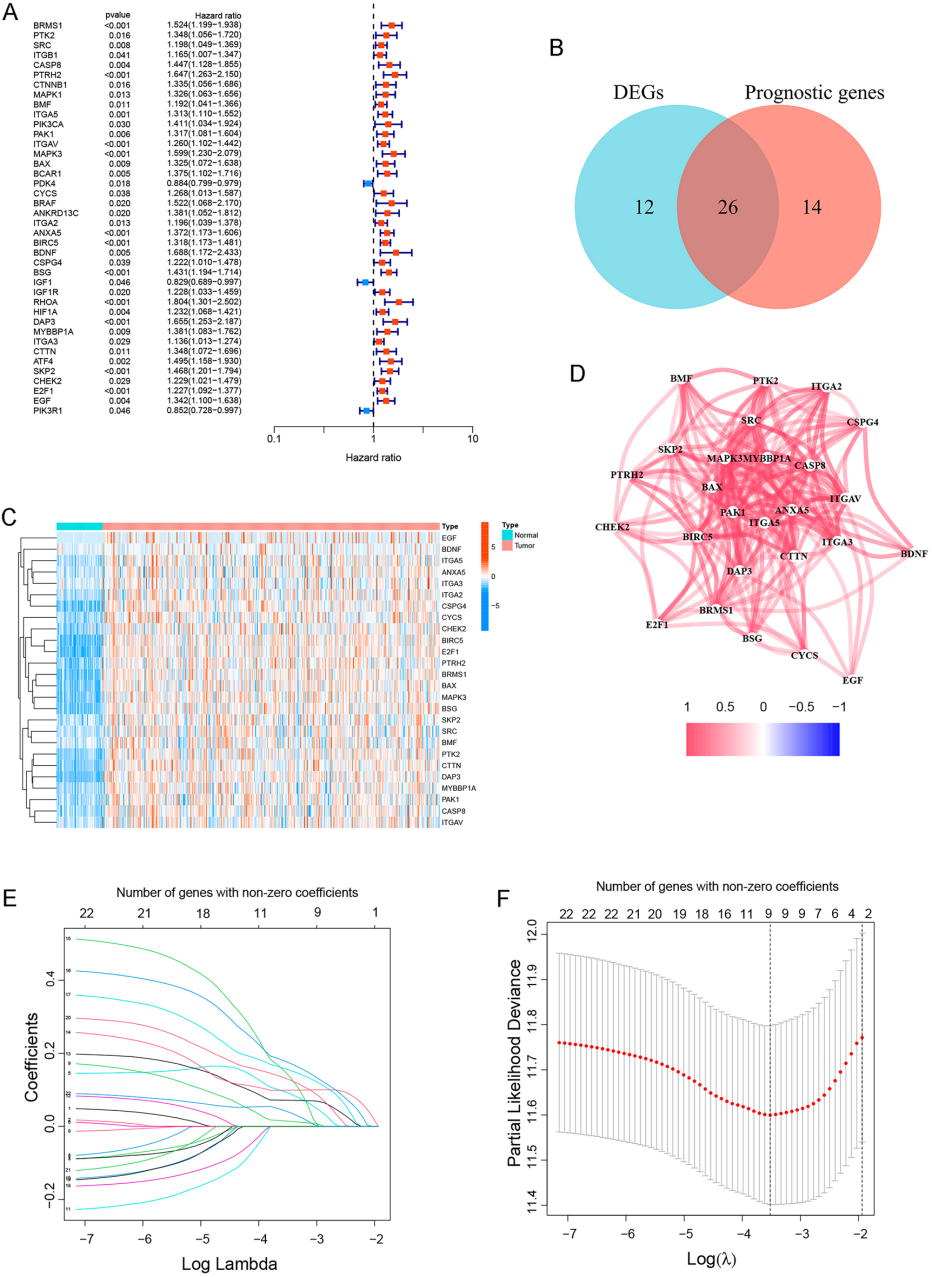
Identify prognostic anoikis-related DEGs and construct the prognostic model in HCC in the TCGA cohort. (**A**) *P*-value and hazard ratio (HR) of 40 anoikis genes related to HCC prognosis by univariate Cox regression analysis. (**B**) Venn diagram showed anoikis-related DEGs and prognostic anoikis-related genes. (**C**) Heatmap showed the expression of 26 overlapping genes between tumor tissues (red) and normal tissues (blue). (**D**) Correlation network of 26 prognostic anoikis-related DEGs. The red lines indicated positive correlations. (**E**) LASSO coefficient profiles of the expression of anoikis-related DEGs. (**F**) Penalty value obtained through LASSO cross-validation. An optimal log λ value was indicated by the vertical black line in the figure.

The general strategy for the present study is shown in Supplementary Fig. S1. We screened anoikis-related genes whose relevance score > 1.8 from the GeneCards and obtained 82 genes (Supplementary Table S1). Subsequently, a total of 40 genes related to HCC prognosis were obtained by univariate Cox regression analysis in the TCGA cohort (Fig. 1A). Among the above 82 genes, 38 genes were DEGs between tumor tissues and normal tissues. We intersected them and finally got 26 prognostic anoikis-related genes (Fig. 1B). Then, we performed the heatmap analysis on the expression of 26 overlapping genes in tumor tissues and normal tissues. In general, the 26 anoikis‑related genes were overexpressed in tumor tissues compared with normal tissues (Fig. 1C). Next, we used the PPI network to find the intrinsic relationship between the 26 genes. In Fig. 1D, the correlation network containing all the anoikis-related genes was presented; PTRH2, ITGAV, ANXA5, BIRC5, BDNF, BSG, DAP3, SKP2 and EGF were identified as hub genes.

### Gene biological function and pathway analysis

We used GO annotation and KEGG pathway enrichment analysis to analyze the biological functions and pathways of the 26 prognostic anoikis-related DEGs. GO enrichment analysis indicated that the 26 DEGs are enriched in biological processes such as anoikis, regulation of anoikis, and regulation of apoptotic signaling pathway. The DEGs mainly have the molecular functions of integrin binding, virus receptor activity, exogenous protein binding, and so on. Besides, they were related to cell components such as focal adhesion and cell-substrate (Supplementary Fig. S2). KEGG pathway analysis revealed that these DEGs were associated with the regulation of human papillomavirus (HPV) infection, focal adhesion, regulation of actin cytoskeleton, and hepatitis (Supplementary Fig. S2). In general, the prognostic anoikis-related DEGs have momentous biological effects in HCC.

### Construction of the prognostic model based on anoikis‑related genes in the TCGA cohort

**Figure 2:**
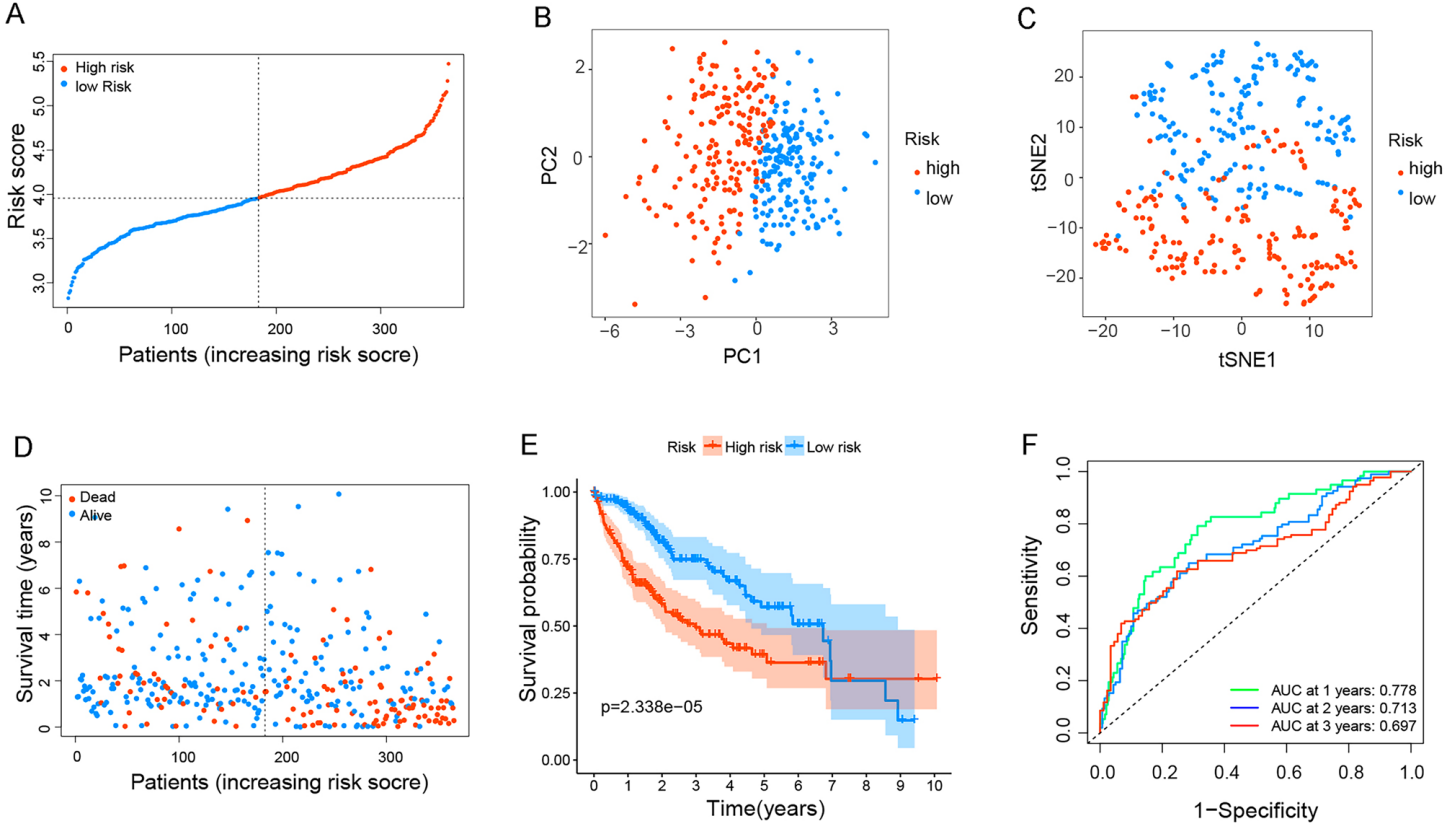
Validation of anoikis prognostic risk model in the TCGA cohort. (**A**–**D**) Risk score (**A**) distribution, (**B**) PCA plot, (**C**) t-SNE, and (**D**) OS status analysis of the TCGA cohort. (**E**) Kaplan–Meier survival curves were used to compare OS between the low-risk and the high-risk groups. (**F**) ROC curves verified the prognostic performance of the risk model.

Subsequently, we developed the risk score model based on 26 prognostic anoikis-related genes (Supplementary Fig. S3). The Lasso coefficient map of 26 candidate gene expression levels was shown in Fig. 1E. The 26 candidate genes were subjected to Lasso regression analysis for dimensionality reduction, and cross-validation was used to obtain the optimal penalty value (λ) in Lasso regression (Fig. 1F). An anoikis prognostic risk score model was established according to the regression coefficient, and the risk score was calculated using the following formula: risk score = 0.093040 × PTRH2 + 0.014600 × ITGAV + 0.071630 × ANXA5 + 0.099028 × BIRC5 + 0.145664 × BDNF + 0.176856 × BSG + 0.156909 × DAP3 + 0.130661 × SKP2 + 0.041248 × EGF. Based on the median risk score calculated by the risk score formula, HCC patients in the TCGA cohort were divided into high-risk or low-risk groups (Fig. 2A). The results of PCA and t-SNE analysis showed that HCC patients in different risk groups were well-separated (Fig. 2B,C). In addition, we further investigated the relationship between survival status, survival time and the risk score. As shown in Fig. 2D, HCC patients presented higher mortality and shorter survival time in the high-risk group than that in the low-risk group. The prognostic significance of this risk model in HCC was further identified utilizing K-M analysis. In Fig. 2E, the OS of the high-risk group was greatly lower than that of the low-risk group (*p* = 2.338e−05). We also used the time-ROC analysis curve to evaluate the sensitivity and specificity of the prognostic risk score model. The area under the receiver operating characteristic curve (AUC) was 0.778 at 1 year, 0.713 at 2 years, and 0.697 at 3 years (Fig. 2F).

### External validation of anoikis prognostic risk model in the ICGC cohort

**Figure 3:**
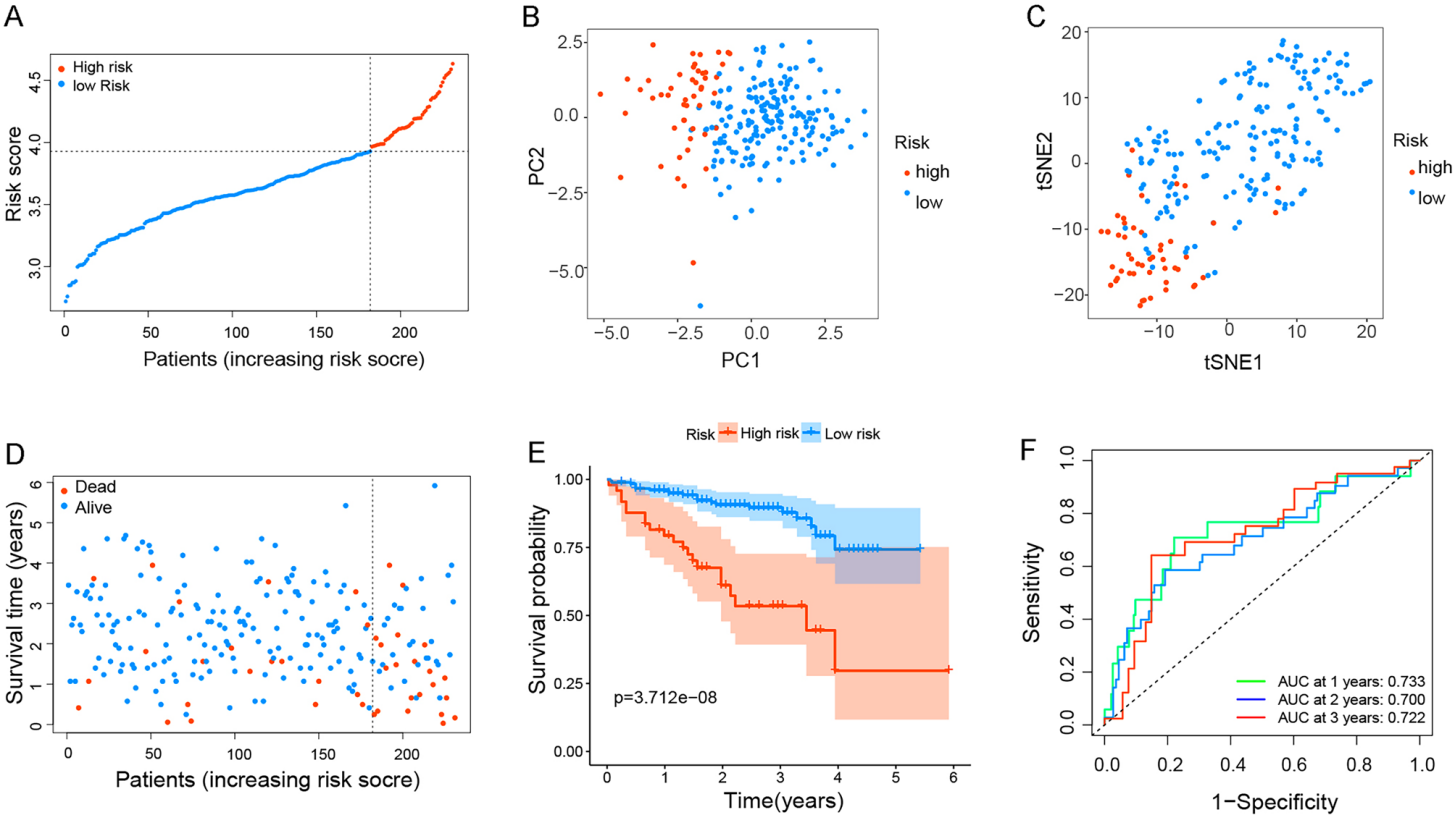
External Validation of anoikis prognostic risk model in the ICGC cohort. (**A**–**D**) Risk score (**A**) distribution, (**B**) PCA plot, (**C**) t-SNE, and (**D**) OS status analysis of the ICGC cohort. (**E**) Kaplan–Meier survival curves were used to compare OS between the low-risk and the high-risk groups. (**F**) ROC curves verified the prognostic performance of the risk model.

To test the reliability of the prognostic risk model established in the TCGA cohort, we selected HCC patients from the ICGC cohort and also divided them into the low-risk group or high-risk group by the median risk score calculated in the TCGA cohort (Fig. 3A). Similarly, PCA and t-SNE analysis exhibited excellent separation between the two groups (Fig. 3B,C). HCC patients in the high-risk group also have a shorter survival time compared with the low-risk group (Fig. 3D). Besides, the K-M survival curve showed the OS of the high-risk group was vastly shorter compared with that in the low-risk group (*p* = 3.712e−08, Fig. 3E). At the same time, the time-ROC curve revealed the strong predictive power of our model in the ICGC cohort, and the AUC predictive values for 1-year, 2-years, and 3-years survival rates were 0.733, 0.700, and 0.722 respectively (Fig. 3F). These results confirmed that our prognostic risk model was reliable and exportable.

### Independent prognostic value of the risk model

**Figure 4:**
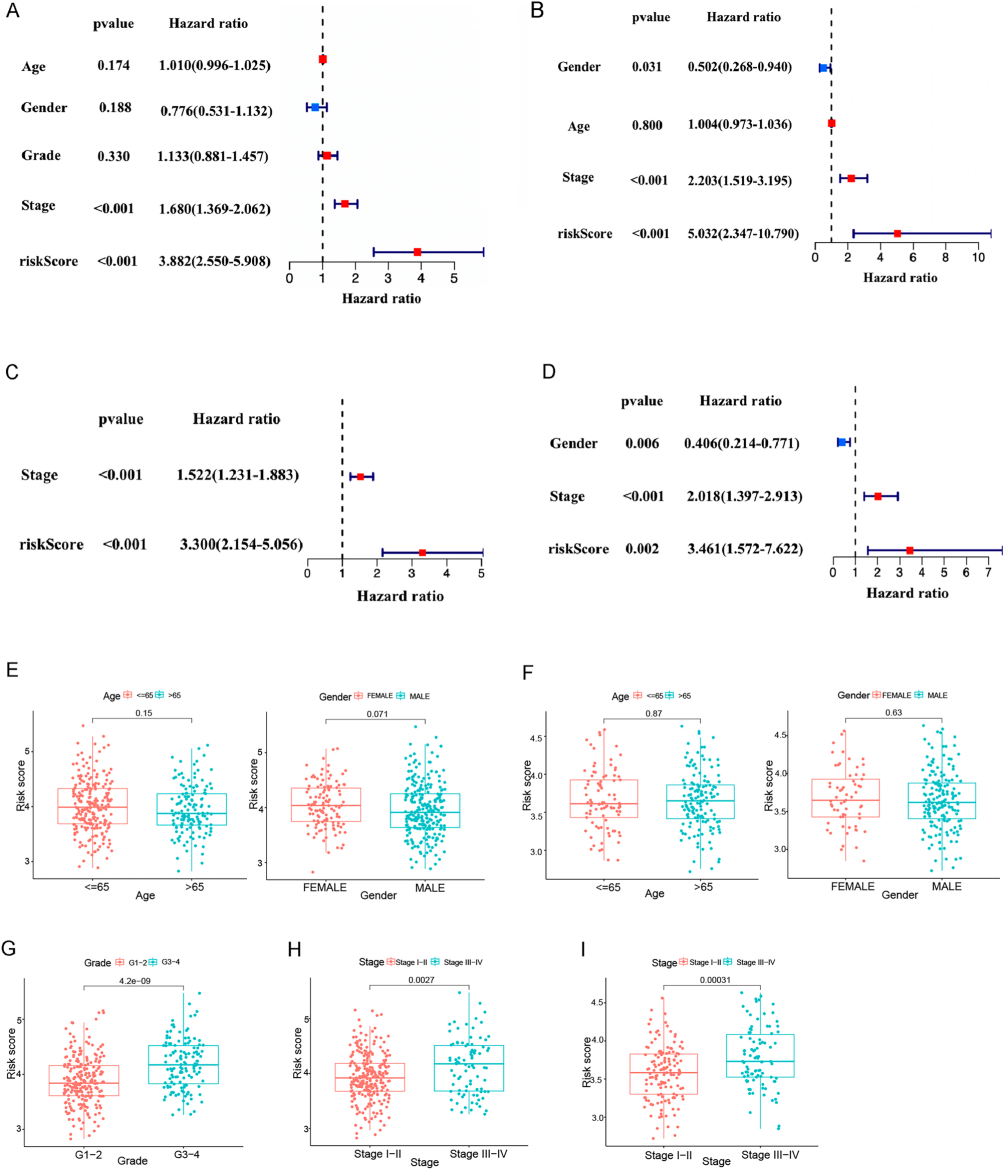
Univariate and multivariate Cox regression analyses for the anoikis risk score and their levels between different clinicopathologic characteristics. (**A**,**B**) Univariate Cox regression analysis of OS in (**A**) the TCGA cohort and (**B**) the ICGC cohort. (**C**,**D**) Multivariate Cox regression analysis of OS in (**C**) the TCGA cohort and the (**D**) ICGC cohort. (**E**–**I**) Anoikis risk score levels of age (≤ 65 and > 65), gender (female and male), groups (group 1–2 and group 3–4), and pathological TNM stages (stage I-II and stage III-IV) in the TCGA cohort (**E**, **G**, and **H**) and the ICGC cohort (**F** and** I**).

To determine whether the risk score could be an independent prognostic indicator, we used the univariate and multivariate Cox regression analysis to evaluate it and other clinical characteristics (age, gender, pathological TNM stage and grade). According to the results of univariate regression analysis, the risk score and pathological TNM stage were significantly correlated with OS of HCC patients in the TCGA cohort (all *p* values < 0.001, Fig. 4A). The risk score, pathological TNM stage, and gender were significantly associated with OS of HCC patients in the ICGC cohort (*p* = 0.031 in gender, and other *p* values < 0.001, Fig. 4B). Furthermore, we conducted the multivariate Cox regression analysis to correct the influencing factors, and the risk score was an independent predictor of OS (TCGA cohort: HR = 3.300, 95% CI = 2.154–5.056, *p* < 0.001; ICGC cohort: HR = 3.461, 95% CI = 1.572–7.622, *p* = 0.002, Fig. 4C,D). In particular, the HR of our risk score was higher than that of the pathological TNM stage.

In addition, we compared the risk score of HCC patients in different clinical characteristic groups. The score was equivalently insignificant between the different age and gender groups (TCGA cohort: *p* = 0.150 and 0.071, Fig. 4E; ICGC cohort: *p* = 0.87 and 0.63, Fig. 4F). In the TCGA cohort, the risk score of grade 3–4 patients was higher than that of grade 1–2 (*p* = 4.2e−09, Fig. 4G). The risk score of pathological TNM III-IV was also found to be higher than that of stage I-II (*p* = 0.0027, Fig. 4H). In the ICGC cohort, the risk score of stage III-IV was also found to be higher than that of stage I-II (*p* = 0.00031, Fig. 4I). These results suggested the high risk score of our model was connected with high-level (grade 3–4) and advanced stage HCC patients.

### Relationship between immune infiltration and anoikis risk score of HCC

**Figure 5:**
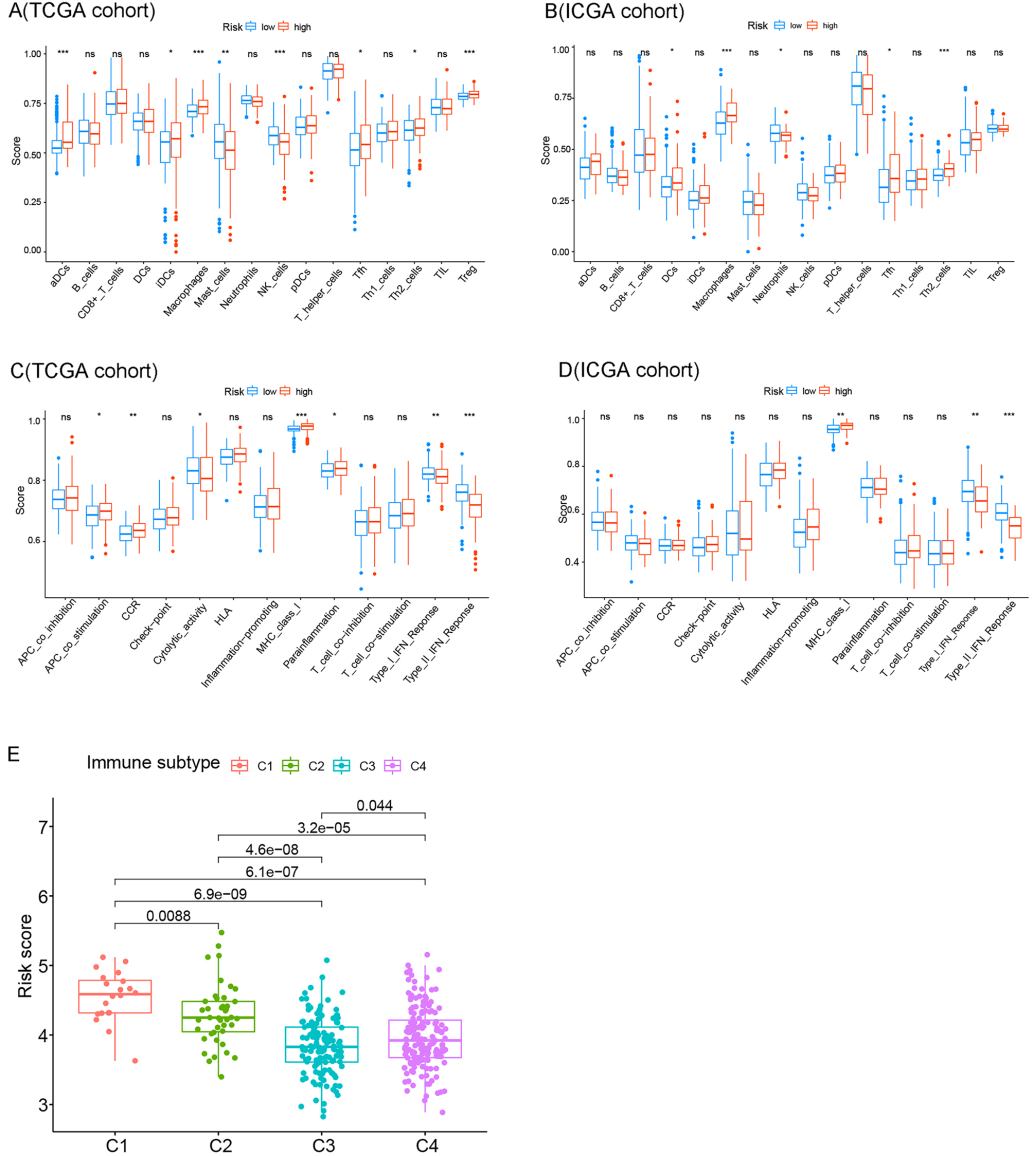
Relationship between immune infiltration and anoikis risk score of HCC. (**A**–**D**) Box plots presented 16 remarkable immune cells and 13 immune-related functions between two groups in the TCGA cohort and the ICGC cohort. The adjusted *p*-value was shown as ns, which is not significant; *p < 0.05; **p < 0.01; and ***P < 0.001. (**E**) Comparison of risk scores in four immune cell subtypes.

To explore the potential clinical relevance between immune status and anoikis, it is worth conducting ssGSEA to calculate the enrichment scores of various immune cells, related functions, or pathways in the TCGA and ICGC cohorts. As shown in Fig. 5A and B, aDCs, macrophages, T-follicular helper cells, and Th2 cells all showed high infiltration in the high-risk groups in the TCGA and ICGC cohorts (*p* < 0.05). Regarding immune-related pathways, MHC class I was significantly upregulated in the high-risk group, while type I IFN response and type II IFN response were downregulated in the TCGA cohort and the ICGC cohort (all adjusted *p* < 0.05, Fig. 5C,D) and the. In addition, we explored the risk scores of the four groups of tumor-infiltrating immune cell subtypes (C1, C2, C3, C4), as shown in Fig. 5E. We found that the highest risk score was closely related to group C1, and the risk score of group C3 was the lowest.

### Correlation between tumor stemness, tumor microenvironment, PD-L1 expression and anoikis risk score

Tumor stemness, including RNAss and DNAss, is a key regulator of tumor progression. DNA methylation-based stem cell index (Manasi) can reflect epigenetic stem cell characteristics, and mRNA expression-based mRNAs represent stem cell expression of the transcriptome. The risk score had no relationship with DNAss, but positively correlated with RNAss (Supplementary Fig. S4A,B). Besides, immune and stromal scores were used to evaluate tumor microenvironment (TME). There was a negative correlation between the risk score and stromal score, but no relationship between the risk score and immune score (Supplementary Fig. S4C,D). The expression level of PD-L1 was significantly higher in the high-risk group compared with the low-risk group (*p* = 0.00048, Supplementary Fig. S4E), and it was positively correlated with the risk score (R = 0.18, *p* = 0.00047, Supplementary Fig. S4F).

### Relationship between the expression of anoikis-related model genes and drug sensitivity

**Figure 6:**
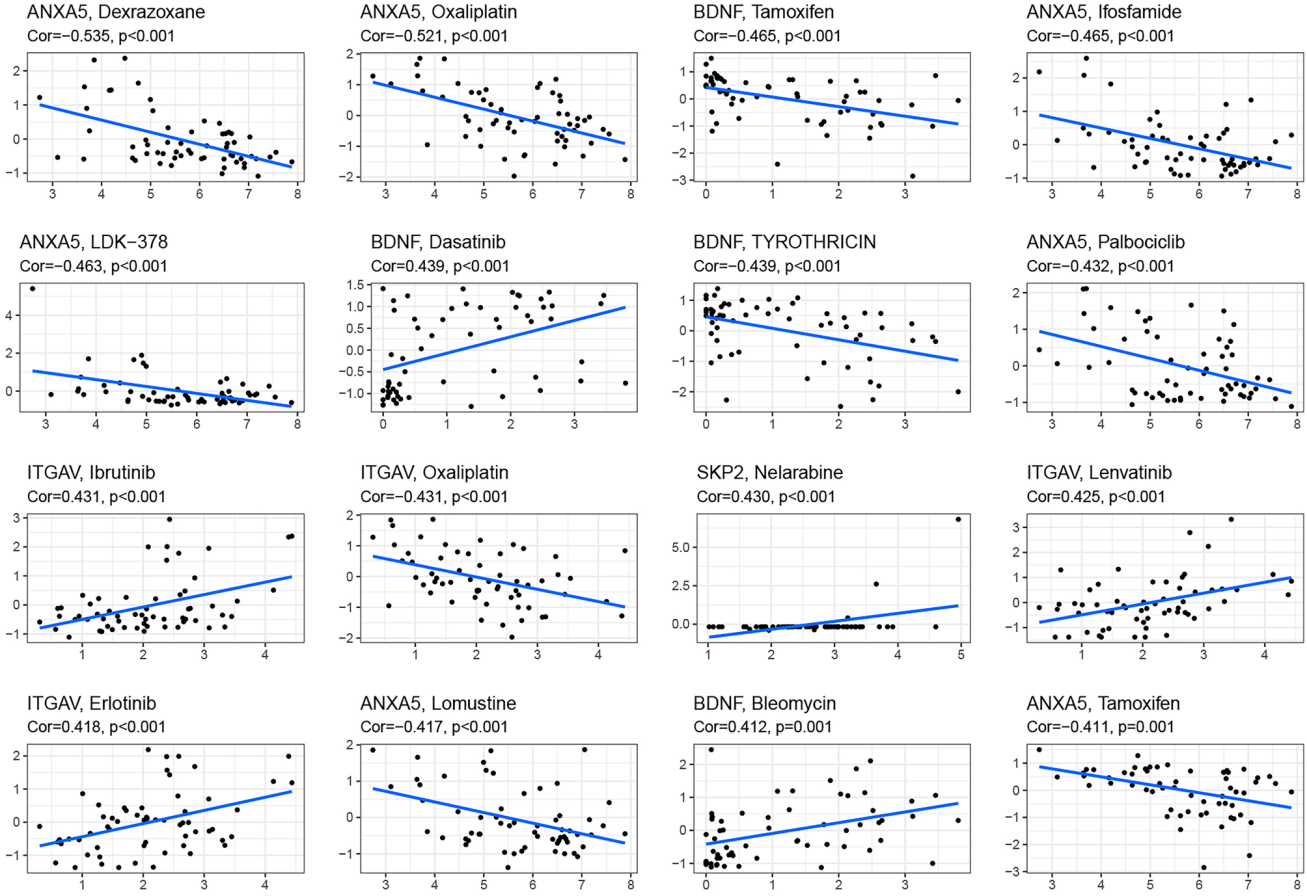
Scatter plots for the correlation between nine model genes expression and drug sensitivity (top 16 pairs ranked by *p* value).

Anoikis-related genes play crucial roles in regulating drug resistance. We used the CellMiner database to explore the relationship between nine model genes and drug sensitivity. The top 16 gene-drug pairs ranked by Pearson correlation coefficient were shown in Fig. 6, which includes Dexrazoxane, LDK-378, Ibrutinib, Erlotinib, Oxaliplatin, Dasatinib, Lomusti, Tamoxifen, Tyrothricin, Nelarabine, Bleomycin, Ifosfamide, Palbociclib and Lenvatinib, respectively. We found that a total of 4 genes were associated with drug sensitivity, namely ANX5, ITGAV, BDNF, and SKP2. SKP2 was positively correlated with the sensitivity of chemotherapy, while ANXA5 was negatively correlated with the sensitivity of targeted drugs. Interestingly, ANXA5 was negatively associated with seven drug sensitivity tests, including chemotherapy drugs and targeted drugs.

### WGCNA identified hub genes in our anoikis model

**Figure 7:**
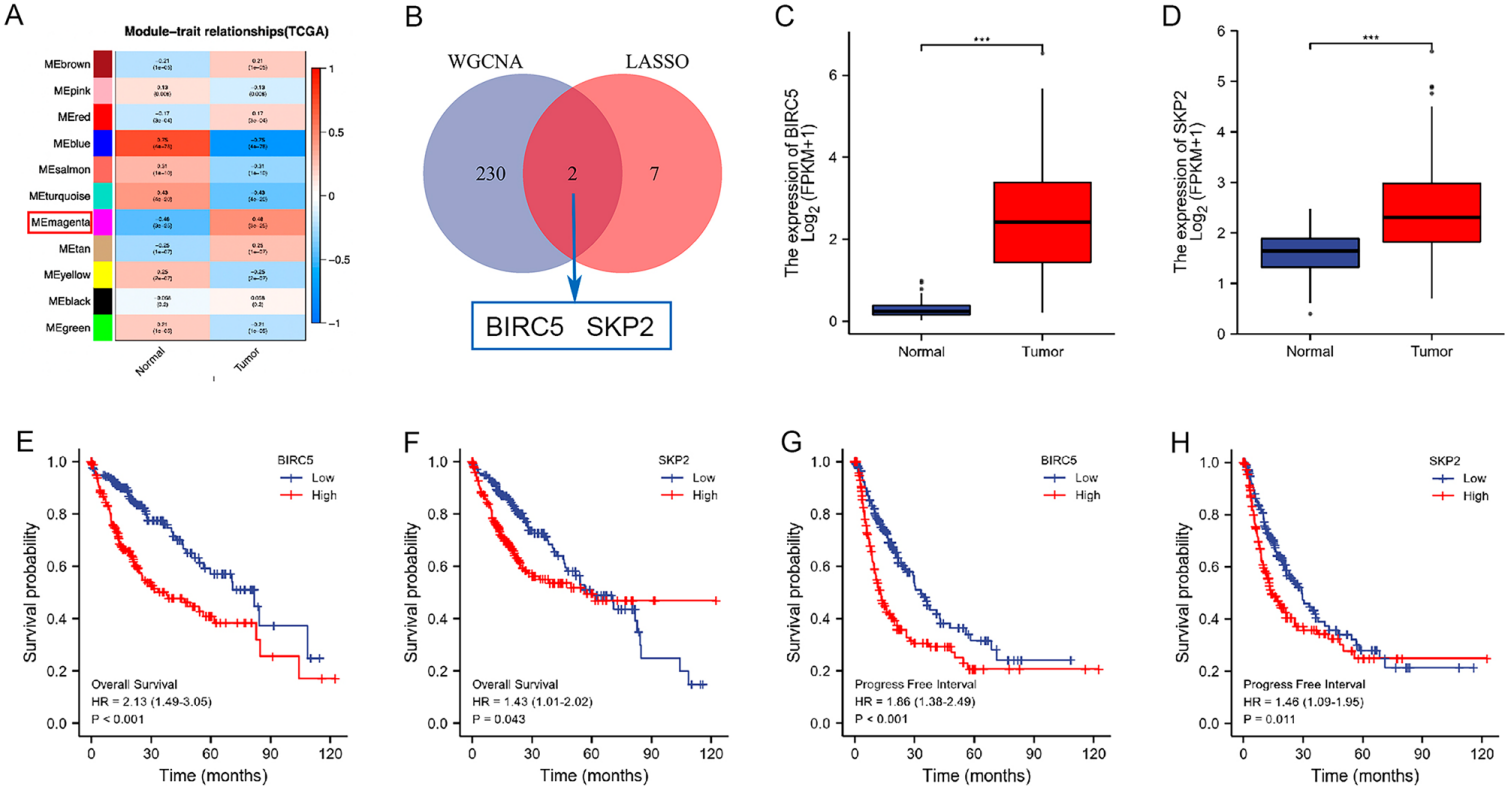
WGCNA identified hub genes in our anoikis model. (**A**) Heatmap of the correlation of gene modules with normal tissues and HCC tissues. (**B**) Venn diagram analysis showed that the overlap of WGCNA analysis and LASSO model led to two hub genes: BIRC5 and SKP2. (**C**,**D**) Differential expression of (**C**) BIRC5 and (**D**) SKP2 in normal tissues and HCC tissues based on TCGA database. (**E**,**F**) K-M curves showing the difference of OS between the (**E**) BIRC5/(**F**) SKP2 high and low expression groups. (**G**,**H**) K-M curves showing the difference of PFI between the (**G**) BIRC5/(**H**) SKP2 high and low expression groups.

To find the most critical genes among the 9 model genes for further study, we performed WGCNA analysis on the TCGA database. Based on the scale-free topological model and average connectivity of the WGCNA analysis, the soft threshold was set to 2 (Supplementary Fig. S5A). The clustered gene dendrogram showed HCC patient co-expression modules, with each leaf representing a gene and branches on the tree representing co-expression modules. (Supplementary Fig. S5B). Eleven correlations were determined by WGCNA analysis between modules and normal samples and HCC, with the pink module having the highest positive correlation in HCC (cor = 0.4, p = 3e−25, Fig. 7A). We identified two hub genes (BIRC5 and SKP2) by intersecting the pink module genes with our model genes through Venn diagrams (Fig. 7B).

Based on the TCGA database, we explored the expression differences of these 2 hub genes between normal tissues and HCC tissues. The results revealed that both BIRC5 and SKP2 were significantly highly expressed in HCC tissues compared to normal tissues (Fig. 7C,D). K-M curves showed that both BIRC5 and SKP2 high expression groups were associated with poor prognosis in patients with HCC compared to low expression groups, both in terms of OS (Fig. 7E,F) and progression-free interval (PFI, Fig. 7G,H).

### Correlation between the expression levels of hub genes and clinicopathological characteristics in the TCGA cohort

The correlation between BIRC5 and SKP2 mRNA expression and clinicopathological characteristics of HCC was assessed based on the TCGA database. Supplementary Table S2 showed that BIRC5 mRNA expression levels were significantly correlated with T-stage, pathologic stage, histologic grade, and Alpha-fetoprotein (AFP). In addition, Supplementary Table S3 displayed that SKP2 mRNA expression levels were significantly correlated with age, histologic grade, and AFP.

Our study further investigated the effects of BIRC5 and SKP2 mRNA expression on OS and PFI in HCC by univariate Cox and multivariate Cox regression analysis. Univariate Cox regression showed that the T stage, M stage, pathologic stage, BIRC5 mRNA expression and SKP2 mRNA expression were correlated with OS in HCC (Supplementary Table S4A). Multivariate Cox regression showed that BIRC5 mRNA expression was an independent factor (Supplementary Table S4B). As for PFI, univariate Cox regression suggested that the T stage, M stage, pathologic stage, vascular invasion, BIRC5 mRNA expression and SKP2 mRNA expression were correlated with it in HCC (Supplementary Table S5A). BIRC5 mRNA expression was an independent factor influencing PFI in HCC (Supplementary Table S5B).

We also analyzed the expression of these two factors in several other cancers, including lung adenocarcinoma (LUAD), breast invasive carcinoma (BRCA), as well as most other digestive tumors, including cholangiocarcinoma (CHOL), colon adenocarcinoma (COAD), esophageal carcinoma (ESCA), pancreatic adenocarcinoma (PAAD) and rectum adenocarcinoma (READ), to verify the specificity of their prognostic value in HCC. The median expression levels in these cancers themselves were used as cutoff. K-M curves showed that only BIRC5 high expression group were associated with poor OS in PAAD, and BIRC5 has no prognostic value in other cancers. SKP2 has no prognostic value in all of the above cancers (Supplementary Fig. S6). So overall, the value of these two factors for poor prognosis in HCC is somewhat specific.

### Immunohistochemical staining of BIRC5 and SKP2 and correlation with clinicopathological characteristics in our TMA cohort

**Figure 8:**
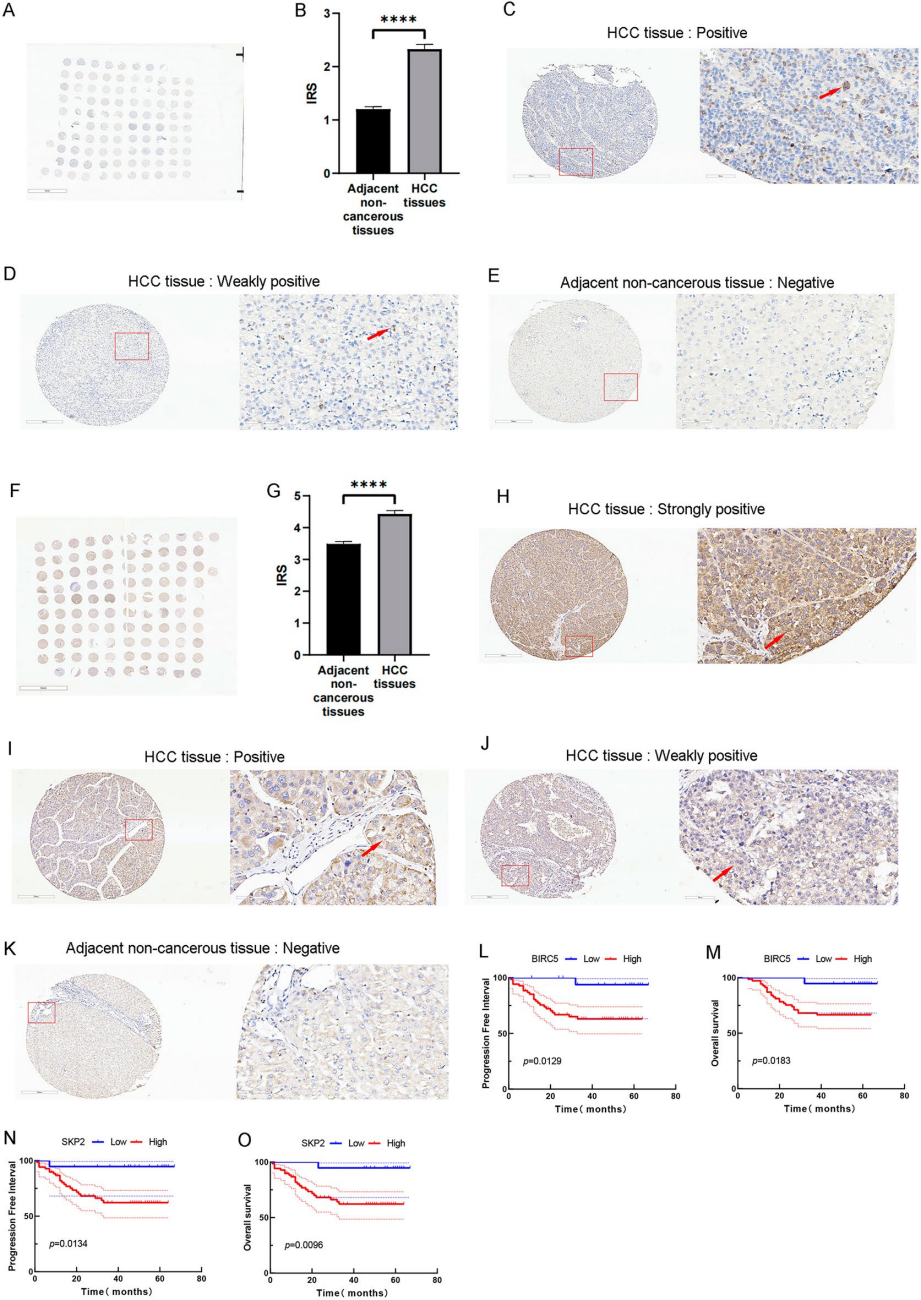
Experimental validation of immunohistochemical staining of BIRC5 and SKP2 in oure TMA cohort and their prognostic value. (**A**) Immunohistochemical staining of BIRC5 in the HCC TMA cohort. (**B**) The IRS of BIRC5 in HCC and adjacent non-cancerous tissues. ****p < 0.0001; Data are presented as the mean ± standard error of mean. (**C**–**E**) The intensities of BIRC5 immunostaining were (**C**) positive, (**D**) weakly positive and (**E**) negative, no strongly staining was observed in HCC tissues. Left image, magnification ×100; right image, magnification ×400; the red squares indicate the area shown in higher magnification. (**F**) Immunohistochemical staining of SKP2 in the HCC TMA cohort. (**G**) The IRS of SKP2 in HCC and adjacent non-cancerous tissues. ****p < 0.0001; Data are presented as the mean ± standard error of mean. (**H**–**K**) The intensities of SKP2 immunostaining were (**H**) strongly positive, (**I**) positive, (**J**) weakly positive and (**K**) negative. Left image, magnification ×100; right image, magnification ×400; the red squares indicate the area shown in higher magnification. (**L**,**M**) Kaplan–Meier survival curves to compare (**L**) OS and (**M**) PFI between the high-BIRC5 expression and low-BIRC5 expression patients. (**N**,**O**) Kaplan–Meier survival curves to compare (**N**) OS and (**O**) PFI between the high-SKP2 expression and low-SKP2 expression patients.

To further experimentally verify the expression levels of BIRC5 and SKP2, 88 HCC and adjacent non-cancerous tissue samples were collected in this study, and the expression levels of BIRC5 and SKP2 were detected by immunohistochemical staining (Fig. 8A,F). BIRC5 was mainly expressed in the nucleus of HCC cells. The IRS of BIRC5 protein was significantly higher in HCC than in adjacent non-cancerous tissues (Fig. 8B–E). In addition, SKP2 was mainly expressed in the cytoplasm of HCC cells. SKP2 protein levels were also significantly higher in HCC tissues than in adjacent non-cancerous tissues (Fig. 8G–K).

We collected detailed information on the clinical characteristics of the TMA cohort to further validate the correlation between BIRC5 or SKP2 expression and clinicopathological characteristics of HCC. Table 1 showed that BIRC5 expression levels were significantly correlated with T-stage, pathologic stage, histologic grade, and AFP. For SKP2, its expression levels were only significantly correlated with age and smoking history (Supplementary Table S6).

**Table 1:** Correlation of BIRC5 expression levels with clinicopathological characteristics of HCC in the TMA cohort.

Characteristic	Low expression of BIRC5	High expression of BIRC5	p
Gender			0.232
Male	13 (68.4%)	56 (81.2%)	
Female	6 (31.6%)	13 (18.8%)	
Age (year)			0.219
≤ 60	8 (42.1%)	40 (58.0%)	
> 60	11 (57.9%)	29 (42.0%)	
Smoking history			0.529
Not	12 (63.2%)	38 (55.1%)	
Have	7 (36.8%)	31 (44.9%)	
Drinking history			0.255
Not	15 (78.9%)	45 (65.2%)	
Have	4 (21.1%)	24 (34.8%)	
Cirrhosis history			0.204
Not	3 (15.8%)	21 (30.4%)	
Have	16 (84.2%)	48 (69.6%)	
T stage			0.032
T1 and T2	19 (100.0%)	55 (79.7%)	
T3 and T4	0 (0.0%)	14 (20.3%)	
N stage			0.453
N0	19 (100.0%)	67 (97.1%)	
N1	0 (0.0%)	2 (2.9%)	
Pathologic stage			0.032
Stage I and II	19 (100.0%)	55 (79.7%)	
Stage III and IV	0 (0.0%)	14 (20.3%)	
AFP			0.028
≤ 6.7 IU/ml	11 (57.9%)	21 (30.4%)	
> 6.7 IU/ml	8 (42.1%)	48 (69.6%)	
TB			0.154
≤ 26 μmol/L	16 (84.2%)	65 (94.2%)	
> 26 μmol/L	3 (15.8%)	4 (5.8%)	
ALB			0.569
< 40 g/L	3 (15.8%)	15 (21.7%)	
≥ 40 g/L	16 (84.2%)	54 (78.3%)	
Hepatits B virus infection			0.673
No	5 (26.3%)	15 (21.7%)	
Yes	14 (73.7%)	55 (78.3%)	
HBV-DNA			0.655
≤ 500 IU/ml	8 (42.1%)	34 (49.3%)	
> 500 IU/ml	4 (21.1%)	17 (24.6%)	
Missing	7 (36.8%)	18 (26.1%)	
Tumor size	4.50 ± 3.23	4.37 ± 2.99	0.873
Microvascular invasion			0.241
Not	10 (52.6%)	26 (37.7%)	
Have	9 (47.4%)	43 (62.3%)	
Satellite nodules			0.152
Not	17 (89.5%)	51 (73.9%)	
Have	2 (10.5%)	18 (26.1%)	
Liver capsule infiltration			0.097
Not	19 (100.0%)	60 (87.0%)	
Have	0 (0.0%)	9 (13.0%)	
Portal vein invasion			0.238
Not	19 (100.0%)	65 (94.2%)	
Have	0 (0.0%)	4 (5.8%)	
Portal vein embolus			0.230
Not	18 (94.7%)	58 (84.1%)	
Have	1 (5.3%)	11 (15.9%)	
Histologic grade			0.024
G1 and G2	17 (89.5%)	43 (62.3%)	
G3 and G4	2 (10.5%)	26 (37.7%)	

In order to further investigate the potential differential expressions of BIRC5 or SKP2 in liver tissues affected by chronic liver diseases such as hepatitis and cirrhosis, as compared to normal liver tissues, we re-classified our original liver control tissues based on the presence or absence of hepatitis and cirrhosis. We first conducted an analysis using Pearson's Chi-squared test and Fisher's exact test. The results revealed that the expression level of BIRC5 was found to be independent of the history of cirrhosis and hepatitis among HCC patients (Table 2A). On the other hand, the expression level of SKP2 showed a significant correlation with the cirrhosis history of HCC patients (p = 0.042), but not with their hepatitis history (Table 2B). However, when examining the IRS scores, no significant difference was observed in the expression of BIRC5 or SKP2 between tissues with or without cirrhosis, as well as between tissues with or without hepatitis (Supplementary Fig. S7). These findings suggest that although there is a higher prevalence of SKP2 positivity among patients with cirrhosis, there are still individuals without cirrhosis who exhibit high SKP2 expression. Consequently, there is no significant difference in the mean values between these two groups. Therefore, further investigation is required to elucidate the role of SKP2 in cirrhosis.

**Table 2:** Correlation of BIRC5 and SKP2 expression levels of liver control tissues with cirrhosis or hepatitis in the TMA cohort.

A. BIRC5 expression levels of liver control tissues in the TMA cohort			
Clinicopathological features	Low expression of BIRC5	High expression of BIRC5	p
Cirrhosis history			0.769
No	20 (28.6%)	4 (22.2%)	
Yes	50 (71.4%)	14 (77.8%)	
Hepatitis history			0.544
No	15 (21.4%)	5 (27.8%)	
Yes	55 (78.6%)	13 (72.2%)	

B. SKP2 expression levels of liver control tissues in the TMA cohort			
Clinicopathological features	Low expression of SKP2	High expression of SKP2	p
Cirrhosis history			0.042
No	14 (38.9%)	10 (19.2%)	
Yes	22 (61.1%)	42 (80.8%)	
Hepatitis history			0.672
No	9 (25.0%)	11 (21.2%)	
Yes	27 (75.0%)	41 (78.8%)	

### Prognostic value of BIRC5 and SKP2 in our TMA cohort

The K-M survival curves of the TMA cohort revealed that high expression of BIRC5 was associated with poor prognosis in HCC (Fig. 8L, PFI, *p* < 0.05; Fig. 8M, OS, *p* < 0.05). Similarly, PFI (*p* < 0.05, Fig. 8N) and OS (*p* < 0.01, Fig. 8O) were significantly lower in SKP2-high expressing HCC compared to SKP2-low expressing HCC. These findings are consistent with the results of our bioinformatics analysis.

Univariate Cox regression showed that T-stage, pathologic stage, histologic grade, BIRC5 expression, and SKP2 expression affected OS in the HCC TMA cohort (Table 3A). Multivariate Cox regression showed that pathologic stage and histologic grade were independent factors affecting OS (the above clinicopathological characteristics and BIRC5 or SKP2 expression, Table 3B). As for PFI, T-stage, pathologic stage, histologic grade, BIRC5 expression, and SKP2 expression were correlated to PFI by univariate Cox regression (Supplementary Table S7A). Multivariate Cox regression showed that the pathologic stage was an independent factor influencing PFI (Supplementary Table S7B).

**Table 3:** Univariate Cox regression analysis and multivariate regression analyses of OS in the TMA cohort.

A. BIRC5 and SKP2 overall survival (OS) univariate analysis					
Characteristics	Total (N)	Univariate analysis			
		Hazard ratio (95% CI)	P value		
Gender (female vs. male)	88	0.465 (0.139–1.560)	0.215		
Age (> 60 vs. ≤ 60)	88	1.344 (0.602–3.000)	0.471		
T stage (T3 and T4 vs. T1 and T2)	88	6.465 (2.841–14.713)	< 0.001		
N stage (N1 vs N0)	88	1.788 (0.241–13.260)	0.570		
Pathological stage (stage III and IV vs. stage I and II)	88	6.465 (2.841–14.713)	< 0.001		
Histologic grade (G3 and G4 vs. G1 and G2)	88	2.783 (1.228–6.106)	0.014		
AFP(IU/ml) (> 6.7 vs. ≤ 6.7)	88	1.896 (0.753–4.778)	0.175		
Vascular invasion (yes vs. no)	88	1.960 (0.812–4.729)	0.135		
BIRC5 (high expression vs. low expression)	88	7.636 (1.031–56.572)	0.047		
SKP2 (high expression vs. low expression)	88	7.481 (1.010–55.413)	0.049		

B. BIRC5 and SKP2 overall survival (OS) multivariate analysis					
Characteristics	Total (N)	BIRC5 Multivariate analysis		SKP2 Multivariate analysis	
		Hazard ratio (95% CI)	P value	Hazard ratio (95% CI)	P value
Pathological stage (stage III and IV vs. stage I and II)	88	6.018 (2.169–16.695)	0.001	6.088 (2.180–16.999)	0.001
Histologic grade G3 and G4 vs. G1 and G2)	88	2.521 (1.085–5.857)	0.032	2.597 (1.100–6.134)	0.029
AFP(IU/ml) (> 6.7 vs. ≤ 6.7)	88	0.931 (0.342–2.535)	0.889	1.344 (0.495–3.650)	0.562
Vascular invasion (yes vs. no)	88	0.835 (0.288–2.415)	0.793	0.727 (0.241–2.192)	0.571
BIRC5/SKP2 (high expression vs. low expression)	88	4.012 (0.508–31.676)	0.188	5.998 (0.788–45.668)	0.084

## Discussion

Given the high heterogeneity observed in HCC patients, it is significant to develop a new prognostic system to predict the outcomes of the patients more accurately. It has been proved that anoikis can block cancer development and resistance in anoikis leads to cancer progression and metastasis. So we obstructed and verified a prognostic risk model based on anoikis-related genes in our study. In addition, we identified BIRC5 and SKP2 as hub genes and preliminarily validated them in the TCGA cohort and our own TMA cohort.

The TNM system, widely applied in the majority of substantial tumors, fails to account for the degree of liver dysfunction and performance status of patients^29^. Although the BCLC system may *p*rovide more prognostic information, it still lacks universal applicability in clinical practices^6^. Under the situation that prognostic systems for HCC patients have certain shortages, several signatures based on hub genes have been established in recent research^30,31^. Here, our model has higher predictive power in contrast to previous studies, for our model was successfully tested in both internal and external databases. Particularly, the time-ROC curve shows that AUC predictive values for the 1-year survival rate are 0.778 in internal validation and 0.733 in external validation, respectively. We noted the AUCs of the prognostic model based on DNA methylation-driven genes for 1 year, 2 years, and 3 years were 0.650, 0.696, and 0.664^32^. Hence, our model is probably more robust and effective. We found that the high-risk score was correlated with adverse clinical characteristics, such as low tumor differentiation and advanced stage, which contribute to poor survival outcomes of high-risk patients. Besides, univariate and multivariate Cox regression analyses revealed that our prognostic signature is capable of serving as an independent prognostic factor for patients with HCC. Furthermore, We not only investigated the role of anoikis in the immune microenvironment but also explored the relationship between anoikis-related DEGs expression and drug sensitivity.

In the present study, we identified a risk model consisting of the nine anoikis-related genes (PTRH2, ITGAV, ANXA5, BIRC5, BDNF, BSG, DAP3, SKP2, and EGF) for predicting the prognosis of HCC. Recently, Chen et al*.* constructed a prognostic anoikis model in HCC using part of the TCGA database as a training dataset^33^. Since we used higher criteria for screening the genes of anoikis (relevance score > 1.8) and the whole TCGA dataset as a training set, only BSG is the same gene among our 9 model genes and the above model. Therefore, our model and their model are completely different. More importantly, we have identified hub genes, BIRC5 and SKP2 in HCC anoikis model using the WGCNA method, which is an innovative finding. Furthermore, we validated the overexpression and prognostic value of these two hub genes in liver cancer through immunohistochemical staining in our cohort, providing further confirmation.

Multiple studies have reported that most of the nine anoikis-related genes are related to cancer. IGTAV, involved in cell–cell and ECM adhesions, serves as an independent prognostic factor in esophageal adenocarcinoma (EAC) without neoadjuvant therapy^34^. In colon cancer cells, activation of TP53 suppresses ITGAV expression, resulting in cell survival^35^. Weiler et al*.* confirmed that overexpression of IGTAV is associated with poor clinical outcomes in patients with HCC^36^. Cilengitide is an ITGAV antagonist, and Mas-Moruno et al*.* identified that cilengitide reduces angiogenesis and inhibits breast cancer bone metastasis in vivo preliminary results^37,38^. Annexin A5 (ANXA5) was reported to participate in HCC pathogenesis via integrin and MEK-ERK pathways^39^. Kang et al*.* suggested that the administration of ANXA5 alleviates the immunosuppressive properties of TME generated by chemotherapy and improves the efficacy of anti-tumor therapy. So the ANXA5 administration following chemotherapy could be considered as a promising immune checkpoint inhibitor for cancer treatment^40^. Basigin (BSG), also known as cluster of differentiation 147 (CD147), is overexpressed and considered to be a prognostic marker in various human tumors^41–45^. It has been observed that CD147 increases cathepsin B expression by activating β-catenin signaling, which mediates CD147-induced invasive phenotype in HCC^46^. Studies have shown that BSG-directed monoclonal antibodies excite clinical success in HCC treatment^47^. Additionally, the overexpression of brain-derived neurotrophic factor (BDNF) and tropomyosin-related kinase B (TrkB) receptors is associated with lower OS in HCC^48^. It was reported that the BDNF/TrkB axis can promote several important biological processes in several types of cancer, such as cell proliferation, tumor immunosuppression, drug resistance, resistance to anoikis, activation of PI3K/AKT and JAK/STAT3 pathways, EMT, and angiogenesis^49^. Several studies have suggested that the downregulation of BDNF suppresses cell viability and metastasis in multiple solid tumors^50,51^. Therefore, the BDNF/TrkB axis can be considered as a potential therapeutic target for several types of cancer. DAP3 was reported to function as a positive mediator of anoikis. Han et al*.* found that the expression of DAP3 is significantly associated with the malignant properties of HCC cells. DAP3 suppresses adenosine-to-inosine (A-to-I) RNA epitome to further promote cancer progression^52^. It was observed that EGF is highly expressed in HCC and positively relates to higher tumor grade. In addition, EGF promotes the migration and aggressiveness of HCC cells mainly via the induction of fibronectin (FN) in vitro^53^.

To identify pivotal genes, we utilized the WGCNA algorithm to screen key genes and acquired the overlapping genes among these key genes and our model genes. We obtained the two hub genes: BIRC5 and SKP2. It is necessary to further investigate the potential mechanisms of the two hub genes.

Survivin (BIRC5), a member of the inhibitor of the apoptosis family, can promote cell division and tumor proliferation^54^. Hori et al*.* found that BIRC can predominantly suppress anoikis^55^. Xu et al*.* suggested that BIRC5 was upregulated in HCC and associated with a poor prognosis^56^. Co-suppression of OCT4 and BIRC5 can inhibit cancer proliferation by inducing cancer cell apoptosis and cell cycle arrest in HCC^57^. Also, the downregulation of BIRC5 leads to the inhibition of migration and invasion in penile cancer^58^. Thereby, BIRC5 should be regarded as a potential target for anti-cancer drugs and prognosis prediction^59^.

S-phase kinase-associated protein 2 (SKP2), also known as p45, is involved in cancer progression, migration, and invasion^60^. High expression of SKP2 was widely observed in various human tumors, indicating the close correlation between tumor progression and SKP2^61–63^. It is reported that the knockdown of the endogenous SKP2 expression by RNA interference reduces cell proliferation, blocks the cell cycle at the G1 phase, and increases apoptosis in HCC^64^. Delogu et al*.* reported that SKP2 cooperates with N-Ras and AKT proto-oncogenes to promote carcinogenesis in HCC^65^. Currently, SKP2 is not only an independent prognostic factor for HCC but also a new target for anti-tumor drugs and gene therapy^52,66^.

We further preliminarily verified the two hub genes based on the TCGA database and HCC tissues we collected. We not only analyzed the mRNA expression of the two hub genes in the TCGA cohort, but also performed immunohistochemical staining to explore the protein expression in our TMA cohort. In the TCGA cohort, we observed the upregulation of the two hub genes in HCC patients, accompanied by poor survival outcomes. The expression level of BIRC5 mRNA was associated with the T stage, pathologic stage, histologic grade and AFP. SKP2 mRNA expression level was closely related to age, histologic grade and AFP. In our TMA cohort, all of the HCC patients have indications for surgery, so the percentage of early-stage patients is more than the TCGA cohort (pathologic stage III & IV is 25.7% in the TCGA cohort, and it is 15.9% in our cohort). Therefore, the OS of HCC patients in our TMA cohort seems relatively longer than that in the TCGA cohort. Besides, HCC tissues and adjacent non-cancerous tissues in our cohort are paired, from the same patients. Even though there are some differences between our patients and the TCGA cohort patients, BIRC5 and SKP2 were still both overexpressed in HCC tissues compared with tumor-adjacent tissues in our TMA cohort. Moreover, high expression of BIRC5 or SKP2 protein also indicated poor prognosis in our TMA cohort. As previously mentioned, Fang et al*.* suggested that silencing of AFP may reduce intracellular BIRC5 mRNA expression to promote apoptosis in HepG2 cells^67^. In lung adenocarcinoma, the high level of BIRC5 was related to advanced AJCC stage, T and N stages^68^. Finally, Taken together, the results above indicated that the expression of the hub genes has a strong correlation with the prognosis and clinical features in HCC, no matter mRNA or protein, which further validated the stability and accuracy of our risk model.

We further employed functional analysis and found that the prognostic model was enriched in anoikis regulation and tumor-related pathways, including PI3K-Akt, ErbB, and P53 pathways. Additionally, our model was significantly associated with virus infection, such as HPV, Human immunodeficiency virus (HIV), Hepatitis B virus (HBV), and Hepatitis C virus (HCV). As revealed by Kakavandi and his colleagues, viral infection promotes resistance to anoikis and provides the opportunity for cancer cells to spread to distant organs, which is in line with our research results^69–71^.

Currently, immunotherapy opens a new chapter for cancer treatment. Investigation of the potential mechanism between immune microenvironment and tumor progression is needed. Studies revealed that macrophages have two extreme functional phenotypes, including M1 and M2 macrophages. Tumor-associated macrophages (TAMs) usually acquire an M2-like phenotype and play vital roles in the TME^72^. TAMs produce several cytokines to suppress other immune cells. Besides, TAMs could actively enhance the remodeling of the tumor stroma and facilitate the metastatic process of cancer cells by producing several proteolytic enzymes^73–75^. In addition, Li et al*.* found that the high level of Th2 cells is significantly correlated with poor prognosis in HCC^76^. In our study, the high-risk group has higher infiltration of macrophages and Th2 cells, which means macrophages and Th2 cells are risky immune cells for HCC. We also discovered that PD-L1 was dramatically upregulated in the high-risk group, pointing out that PD-L1 inhibitors could be more effective in HCC patients in the high-risk group. Besides, it is important to consider the contributions of BIRC5 and SKP2 to the tumor microenvironment. BIRC5 is a potential biomarker and inducer of myeloid-derived suppressor cell (MDSC) infiltration in HCC, leading to T cell rejection or dysfunction of the tumor immune microenvironment, ultimately reducing the response to immune checkpoint inhibitors (ICIs)^77^. Cytotoxic T lymphocytes (CTLs) are the primary lymphocytes responsible for killing cancer cells in liver cancer^78^. They primarily restrict tumor growth by inducing G1 phase cell cycle arrest, a mechanism that may involve downregulation of SKP2^79^. Additionally, SKP2 knockdown has been found to significantly increase the expression of immunoinfiltration-related genes in osteosarcoma (OS) mouse models, suggesting that SKP2 may mediate immune rejection in the tumor microenvironment^80^.

Resistance of cancer cells to chemotherapy remains a big challenge in cancer treatment. Resistance to cisplatin treatment is a major clinical issue in HCC^81^. Studies have demonstrated that suppressing BIRC5 can enhance the cytotoxicity of chemotherapy agents like cisplatin^82^. Asechi et al. discovered that BIRC5 expression, mediated by PI3K, enables rat hepatoma cell lines to resist cisplatin-induced apoptosis^83^. Sorafenib, the first-line chemotherapy agent for advanced HCC, sensitizes resistant HCC cells to radiation-induced apoptosis by inhibiting STAT3 phosphorylation and reducing BIRC5 expression^84^. Furthermore, the combination of Sorafenib (SF) and BIRC5 shRNA has shown a synergistic effect in reversing multidrug resistance (MDR). Regarding SKP2, studies have reported that both lonafarnib and troglitazone induce G1-S phase block by down-regulating SKP2. Conversely, ectopic overexpression of SKP2 leads to resistance to troglitazone, resulting in a decrease in the number of G1-phase blocked cells^85,86^. Hepatitis B virus X has been found to downregulate SHIP, promoting HCC metastasis and chemical resistance by inducing SKP2 expression^87^. Oxaliplatin (OXA) is one of the most common chemotherapy drugs for patients with HCC^88^. In HCC, cyanidin significantly increases OXA sensitivity and inhibits the EMT induced by OXA via PI3K/Akt signaling^89^. In addition, PKI-587, the dual PI3K/mTOR inhibitor, increases the chemosensitivity of OXA in HCC^90^. In our study, the high level of ANXA5 and ITGAV is negatively related to the low sensitivity to OXA.The underlying mechanism between low sensitivity to OXA and ANXA5 and ITGAV needs further exploration. Currently, Lenvatinib is considered a new first-line targeted therapy for patients with advanced HCC. In our study, high expression of ITGAV is positively related to the high sensitivity of lenvatinib, which indicates that HCC patients with high expression of ITGAV could benefit from lenvatinib. In summary, the combination of specific target inhibitors and chemotherapy drugs appears to be a promising option for the treatment of HCC.

However, a few limitations of this study should be taken into consideration. First, the construction and validation of our prognostic model are based on retrospective databases, so our results should be further confirmed by prospective clinical research. Second, further experimental studies of the nine anoikis-related genes in HCC progression are needed.

## Conclusions

Our study established a novel risk signature based on nine anoikis-related genes, which perform a good capability in predicting the prognosis of HCC patients. In addition, our study reflected that risk score was closely related to the immune microenvironment and analyzed the relationship between anoikis-related genes and drug resistance. Finally, we identified BIRC5 and SKP2 as hub genes among the nine model genes, and verified them at the mRNA and protein levels. The results confirmed that BIRC5 and SKP2 should be considered as potential prognostic predictors in HCC. Altogether, our study can not only provide potential therapeutic targets for HCC but also bring new insights into drug resistance and ameliorate the effect of HCC treatment.

### Supplementary Information

Supplementary Figure S1.

Supplementary Figure S2.

Supplementary Figure S3.

Supplementary Figure S4.

Supplementary Figure S5.

Supplementary Figure S6.

Supplementary Figure S7.

Supplementary Table S1.

Supplementary Table S2.

Supplementary Table S3.

Supplementary Table S4.

Supplementary Table S5.

Supplementary Table S6.

Supplementary Table S7.

## Data Availability

The datasets generated and/or analysed during the current study are available in the TCGA repository (https://portal.gdc.cancer.gov/) and ICGC repository (https://dcc.icgc.org/).
