# Supplementary figures and images for: Development of a prognostic model for anoikis and identifies hub genes in hepatocellular carcinoma
Source: Sci Rep. 2023 Sep 7;13:14723. doi: 10.1038/s41598-023-41139-9 (PMC10484901; doi:10.1038/s41598-023-41139-9)

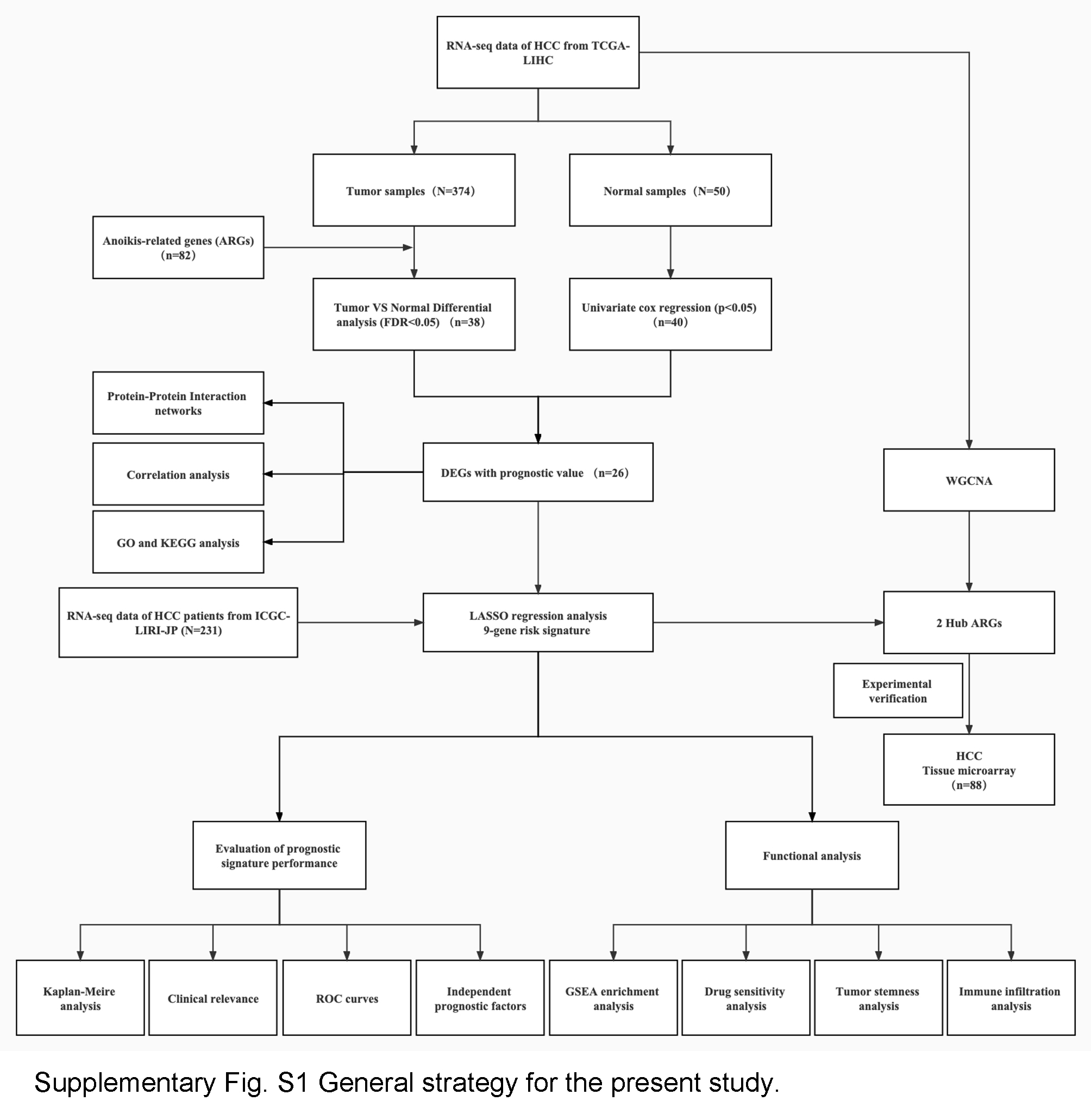

Supplement: Supplementary file 1 — Supplementary Figure S1. [file 41598_2023_41139_MOESM1_ESM.jpg]

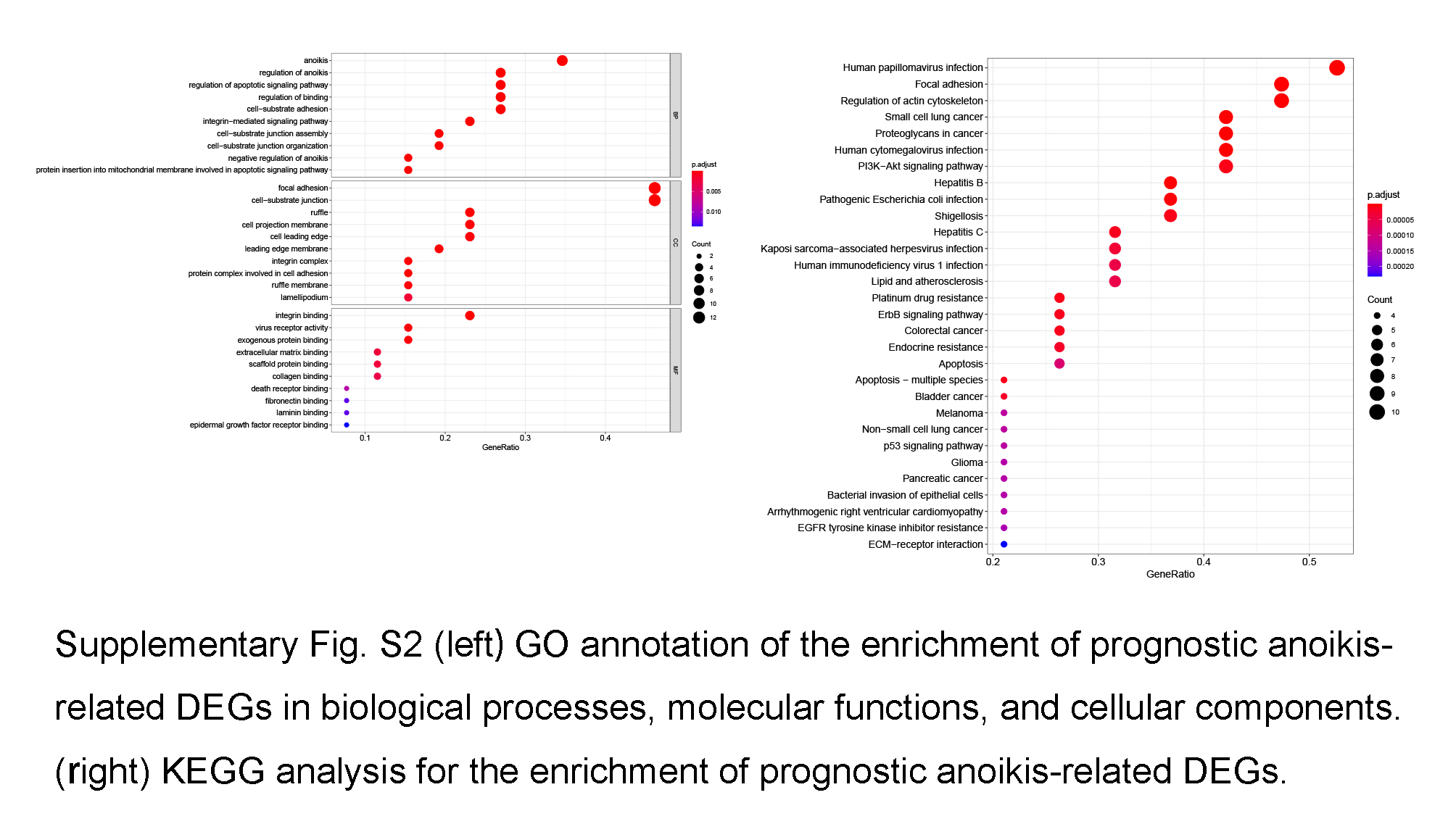

Supplement: Supplementary file 2 — Supplementary Figure S2. [file 41598_2023_41139_MOESM2_ESM.jpg]

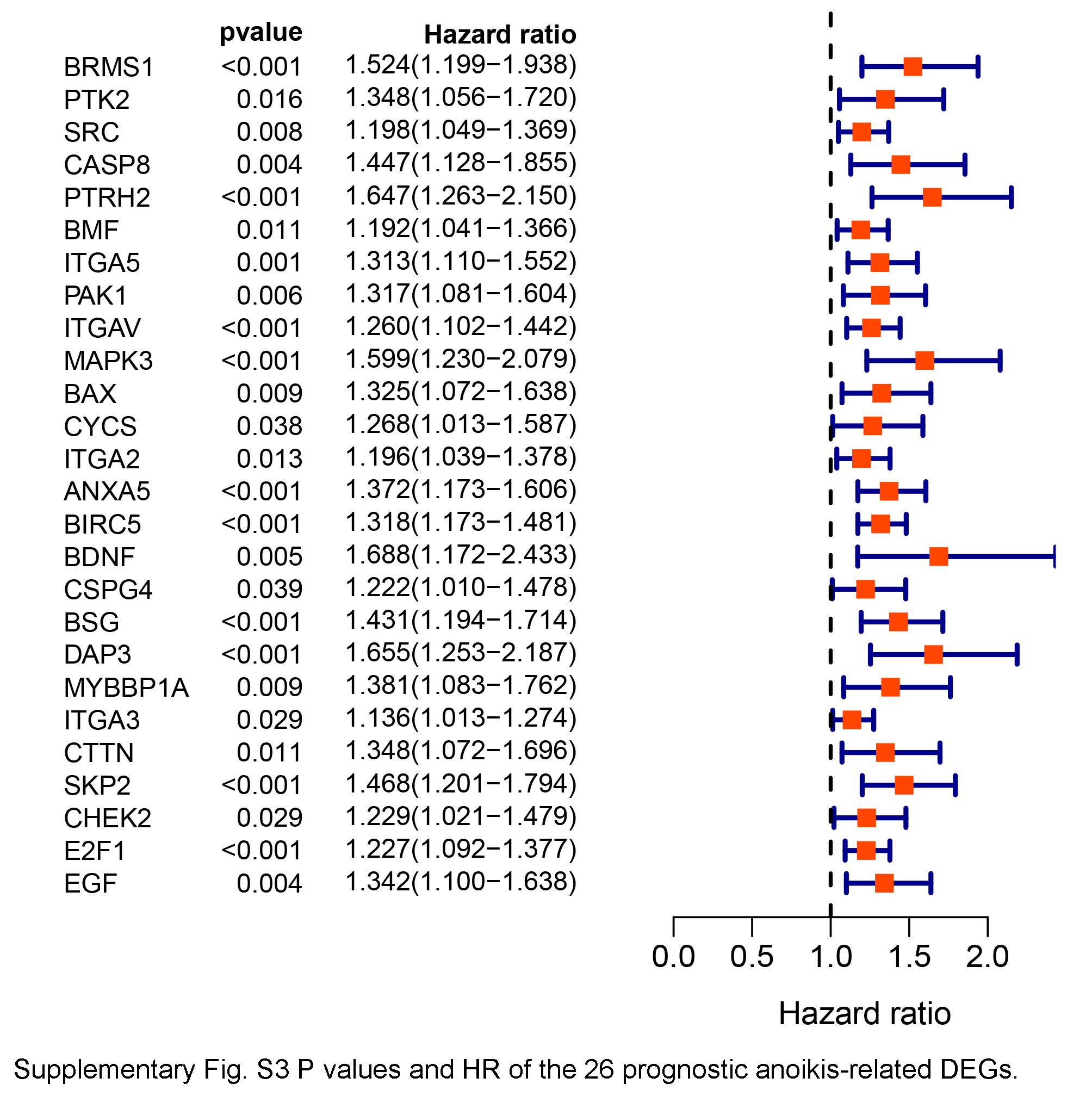

Supplement: Supplementary file 3 — Supplementary Figure S3. [file 41598_2023_41139_MOESM3_ESM.jpg]

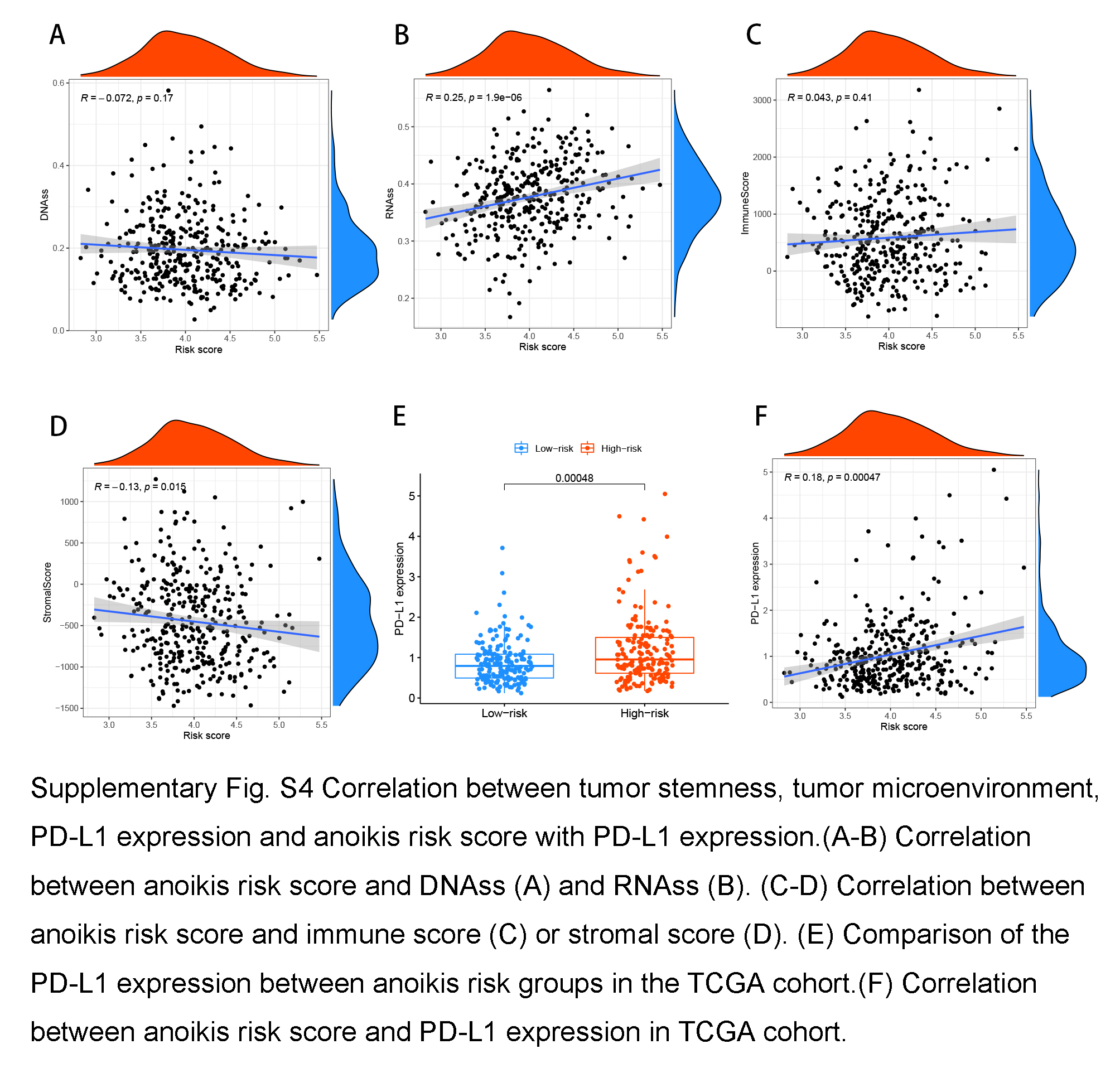

Supplement: Supplementary file 4 — Supplementary Figure S4. [file 41598_2023_41139_MOESM4_ESM.jpg]

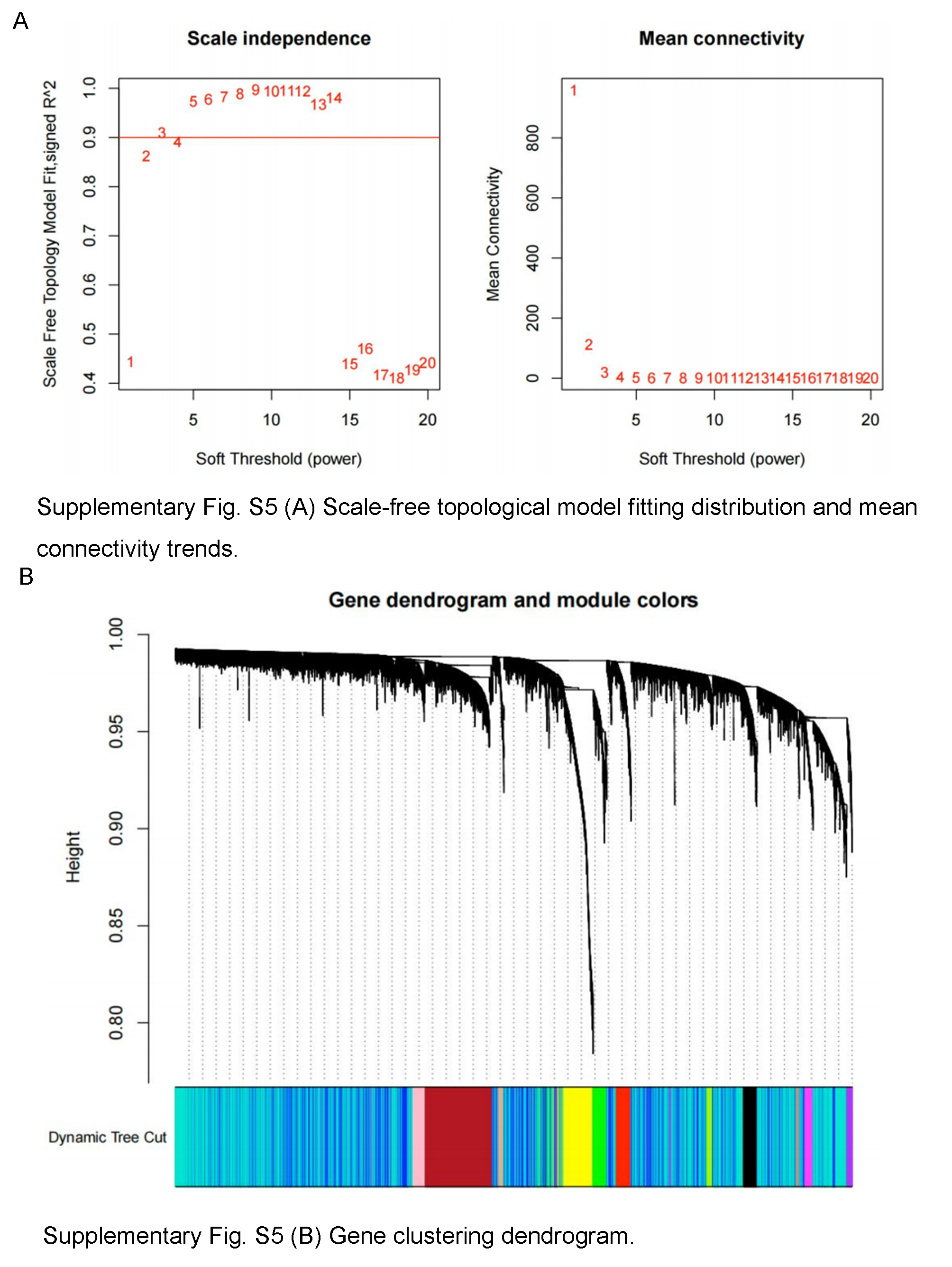

Supplement: Supplementary file 5 — Supplementary Figure S5. [file 41598_2023_41139_MOESM5_ESM.jpg]

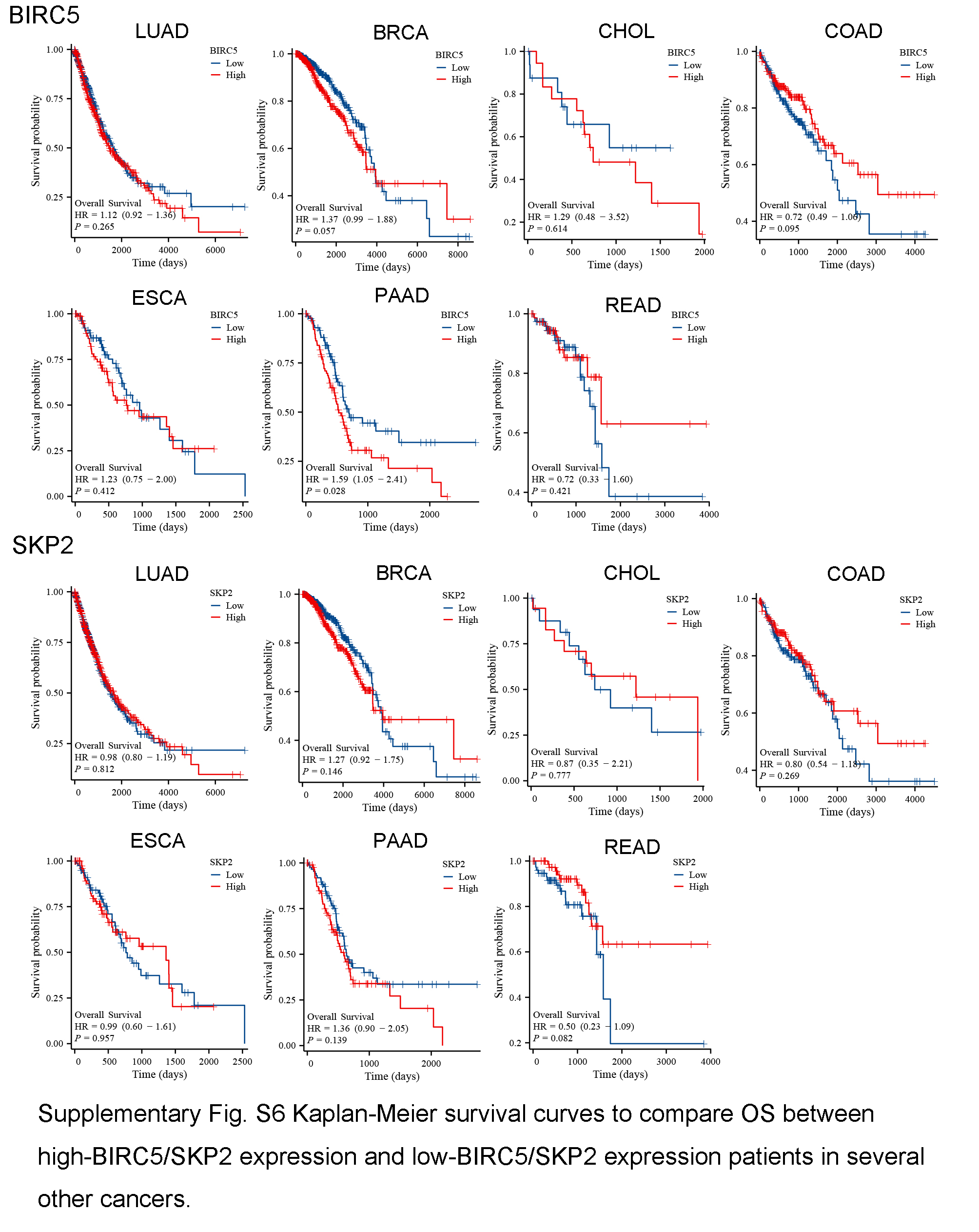

Supplement: Supplementary file 6 — Supplementary Figure S6. [file 41598_2023_41139_MOESM6_ESM.jpg]

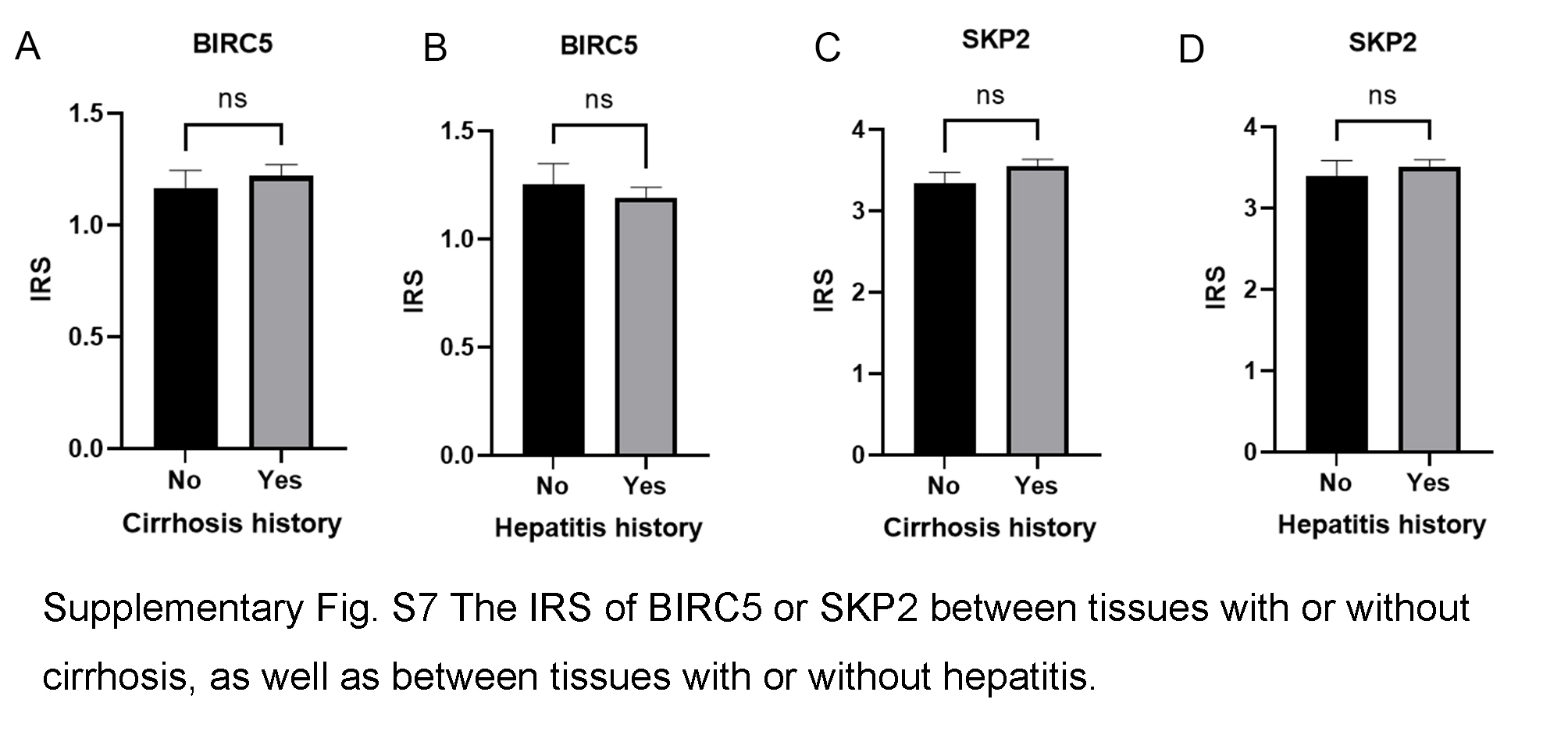

Supplement: Supplementary file 7 — Supplementary Figure S7. [file 41598_2023_41139_MOESM7_ESM.jpg]
