# Supplementary material for: Development of a prognostic model for anoikis and identifies hub genes in hepatocellular carcinoma
Source: Sci Rep. 2023 Sep 7;13:14723. doi: 10.1038/s41598-023-41139-9 (PMC10484901; doi:10.1038/s41598-023-41139-9)
Supplement: Supplementary file 9 — Supplementary Table S2. [file 41598_2023_41139_MOESM9_ESM.docx]

**Supplementary Table S2. Correlation between BIRC5 expression level and clinicopathological characteristics of HCC in the TCGA cohort.**

| **Characteristic** | **Low expression of BIRC5** | **High expression of BIRC5** | **p** |
| --- | --- | --- | --- |
| Gender, n (%) |  |  | 0.269 |
| Female | 55 (29.4%) | 66 (35.3%) |  |
| Male | 132 (70.6%) | 121 (64.7%) |  |
| Age, n (%) |  |  | 0.196 |
| ≤60 | 82 (43.9%) | 95 (51.1%) |  |
| >60 | 105 (56.1%) | 91 (48.9%) |  |
| T stage, n (%) |  |  | **0.004** |
| T1&T2 | 150 (81.5%) | 128 (68.4%) |  |
| T3&T4 | 34 (18.5%) | 59 (31.6%) |  |
| N stage, n (%) |  |  | 1.000 |
| N0 | 120 (98.4%) | 134 (98.5%) |  |
| N1 | 2 (1.6%) | 2 (1.5%) |  |
| M stage, n (%) |  |  | 1.000 |
| M0 | 130 (98.5%) | 138 (98.6%) |  |
| M1 | 2 (1.5%) | 2 (1.4%) |  |
| Pathologic stage, n (%) |  |  | **0.007** |
| Stage I&Stage II | 141 (80.6%) | 119 (68.0%) |  |
| Stage III&Stage IV | 34 (19.4%) | 56 (32.0%) |  |
| Histologic grade, n (%) |  |  | **< 0.001** |
| G1&G2 | 136 (73.5%) | 97 (52.7%) |  |
| G3&G4 | 49 (26.5%) | 87 (47.3%) |  |
| AFP(ng/ml), n (%) |  |  | **< 0.001** |
| ≤400 | 127 (87.0%) | 88 (65.7%) |  |
| >400 | 19 (13.0%) | 46 (34.3%) |  |
| Vascular invasion, n (%) |  |  | 0.309 |
| No | 116 (68.2%) | 92 (62.2%) |  |
| Yes | 54 (31.8%) | 56 (37.8%) |  |
| Age, median (IQR) | 63 (53.5, 69.5) | 60 (51, 68) | 0.072 |
