# Supplementary material for: Development of a prognostic model for anoikis and identifies hub genes in hepatocellular carcinoma
Source: Sci Rep. 2023 Sep 7;13:14723. doi: 10.1038/s41598-023-41139-9 (PMC10484901; doi:10.1038/s41598-023-41139-9)
Supplement: Supplementary file 10 — Supplementary Table S3. [file 41598_2023_41139_MOESM10_ESM.docx]

**Supplementary Table S3.** Correlation between SKP2 expression levels and clinicopathological characteristics of HCC in the TCGA cohort.

| **Characteristic** | **Low expression of SKP2** | **High expression of SKP2** | **p** |
| --- | --- | --- | --- |
| Gender, n (%) |  |  | 0.507 |
| Female | 57 (30.5%) | 64 (34.2%) |  |
| Male | 130 (69.5%) | 123 (65.8%) |  |
| Age, n (%) |  |  | **0.026** |
| ≤60 | 77 (41.4%) | 100 (53.5%) |  |
| >60 | 109 (58.6%) | 87 (46.5%) |  |
| T stage, n (%) |  |  | 0.220 |
| T1&T2 | 143 (77.7%) | 135 (72.2%) |  |
| T3&T4 | 41 (22.3%) | 52 (27.8%) |  |
| N stage, n (%) |  |  | 1.000 |
| N0 | 115 (98.3%) | 139 (98.6%) |  |
| N1 | 2 (1.7%) | 2 (1.4%) |  |
| M stage, n (%) |  |  | 1.000 |
| M0 | 129 (98.5%) | 139 (98.6%) |  |
| M1 | 2 (1.5%) | 2 (1.4%) |  |
| Pathologic stage, n (%) |  |  | 0.246 |
| Stage I&Stage II | 134 (77.0%) | 126 (71.6%) |  |
| Stage III&Stage IV | 40 (23.0%) | 50 (28.4%) |  |
| Histologic grade, n (%) |  |  | **< 0.001** |
| G1&G2 | 137 (74.5%) | 96 (51.9%) |  |
| G3&G4 | 47 (25.5%) | 89 (48.1%) |  |
| AFP(ng/ml), n (%) |  |  | **< 0.001** |
| ≤400 | 135 (91.2%) | 80 (60.6%) |  |
| >400 | 13 (8.8%) | 52 (39.4%) |  |
| Vascular invasion, n (%) |  |  | 1.000 |
| No | 107 (65.6%) | 101 (65.2%) |  |
| Yes | 56 (34.4%) | 54 (34.8%) |  |
| Age, median (IQR) | 64 (54.25, 70) | 59 (50, 66) | 0.001 |
