# Supplementary material for: Development of a prognostic model for anoikis and identifies hub genes in hepatocellular carcinoma
Source: Sci Rep. 2023 Sep 7;13:14723. doi: 10.1038/s41598-023-41139-9 (PMC10484901; doi:10.1038/s41598-023-41139-9)
Supplement: Supplementary file 11 — Supplementary Table S4. [file 41598_2023_41139_MOESM11_ESM.docx]

**Supplementary Table S4**. Univariate Cox regression analysis and multivariate regression analyses of OS in the TCGA cohort

| 1. **BIRC5 and SKP2 overall survival（OS）univariate analysis** | | | | | | | | | |
| --- | --- | --- | --- | --- | --- | --- | --- | --- | --- |
| **Characteristics** | | | **Total(N)** | **Univariate analysis** | | | | | |
|  |  |  |  | **Hazard ratio (95% CI)** | | | **P value** | | |
| Gender（Female vs.Male） | | | 373 | 0.793 (0.557-1.130) | | | 0.200 | | |
| Age (>60 vs. ≤60 ) | | | 373 | 0.830 (0.585-1.176) | | | 0.295 | | |
| T stage (T3&T4 vs. T1&T2) | | | 370 | 0.385 (0.271-0.548) | | | **<0.001** | | |
| N stage (N1 vs. N0) | | | 258 | 0.493 (0.121-2.012) | | | 0.324 | | |
| M stage (M1 vs. M0) | | | 272 | 0.245 (0.077-0.781) | | | **0.017** | | |
| Pathologic stage (Stage III&IV vs. Stage I&Stage II) | | | 349 | 0.399 (0.275-0.579) | | | **<0.001** | | |
| Histologic grade (G3&G4 vs. G1&G2) | | | 368 | 0.917 (0.639-1.314) | | | 0.636 | | |
| AFP(ng/ml) (>400 vs. ≤400) | | | 279 | 0.930 (0.569-1.521) | | | 0.772 | | |
| Vascular invasion (Yes vs. No) | | | 317 | 0.744 (0.491-1.127) | | | 0.163 | | |
| BIRC5 (High expression vs. Low expression) | | | 373 | 0.469 (0.328-0.672) | | | **<0.001** | | |
| SKP2 (High expression vs. Low expression) | | | 373 | 0.699 (0.494-0.989) | | | **0.043** | | |
| 1. **BIRC5 and SKP2 overall survival（OS） multivariate analysis** | | | | | | | | |  |
| **Characteristics** | **Total(N)** | **BIRC5 Multivariate analysis** | | | | **SKP2 Multivariate analysis** | | |  |
|  |  | **Hazard ratio (95% CI)** | | | **P value** | **Hazard ratio (95% CI)** | | **P value** |  |
| T stage (T3&T4 vs. T1&T2) | 370 | 0.710 (0.097-5.211) | | | 0.736 | 0.663 (0.090-4.860) | | 0.686 |  |
| M stage (M1 vs. M0) | 272 | 0.444 (0.136-1.453) | | | 0.180 | 0.448 (0.137-1.468) | | 0.185 |  |
| Pathologic stage (Stage III&IV vs. Stage I&Stage II) | 349 | 0.594 (0.081-4.327) | | | 0.607 | 0.561 (0.077-4.087) | | 0.568 |  |
| BIRC5/SKP2 (High expression vs. Low expression) | 373 | 0.413 (0.257-0.664) | | | **<0.001** | 0.713 (0.462-1.103) | | 0.128 |  |
