# Supplementary material for: Development of a prognostic model for anoikis and identifies hub genes in hepatocellular carcinoma
Source: Sci Rep. 2023 Sep 7;13:14723. doi: 10.1038/s41598-023-41139-9 (PMC10484901; doi:10.1038/s41598-023-41139-9)
Supplement: Supplementary file 12 — Supplementary Table S5. [file 41598_2023_41139_MOESM12_ESM.docx]

**Supplementary Table S5**. Univariate Cox regression analysis and multivariate regression analyses of PFI in the TCGA cohort

| 1. **BIRC5 and SKP2 progression-free interval（PFI）univariate analysis** | | | | | | | | | |
| --- | --- | --- | --- | --- | --- | --- | --- | --- | --- |
| **Characteristics** | | | **Total(N)** | **Univariate analysis** | | | | | |
|  |  |  |  | **Hazard ratio (95% CI)** | | | **P value** | | |
| Gender（Female vs.Male） | | | 373 | 0.982 (0.721-1.338) | | | 0.909 | | |
| Age (>60 vs. ≤60 ) | | | 373 | 1.042 (0.779-1.393) | | | 0.783 | | |
| T stage (T3&T4 vs. T1&T2) | | | 370 | 0.459 (0.336-0.629) | | | **<0.001** | | |
| N stage (N1 vs. N0) | | | 258 | 0.730 (0.180-2.956) | | | 0.659 | | |
| M stage (M1 vs. M0) | | | 272 | 0.288 (0.090-0.917) | | | **0.035** | | |
| Pathologic stage (Stage III&IV vs. Stage I&Stage II) | | | 349 | 0.454 (0.328-0.629) | | | **<0.001** | | |
| Histologic grade (G3&G4 vs. G1&G2) | | | 368 | 0.868 (0.642-1.172) | | | 0.355 | | |
| AFP(ng/ml) (>400 vs. ≤400) | | | 279 | 0.957 (0.640-1.432) | | | 0.832 | | |
| Vascular invasion (Yes vs. No) | | | 317 | 0.597 (0.426-0.836) | | | **0.003** | | |
| BIRC5 (High expression vs. Low expression) | | | 373 | 0.539 (0.401-0.723) | | | **<0.001** | | |
| SKP2 (High expression vs. Low expression) | | | 373 | 0.687 (0.513-0.919) | | | **0.011** | | |
| 1. **BIRC5 and SKP2 progression-free interval（PFI） multivariate analysis** | | | | | | | | |  |
| **Characteristics** | **Total(N)** | **BIRC5 Multivariate analysis** | | | | **SKP2 Multivariate analysis** | | |  |
|  |  | **Hazard ratio (95% CI)** | | | **P value** | **Hazard ratio (95% CI)** | | **P value** |  |
| T stage (T3&T4 vs. T1&T2) | 370 | 2.501 (0.536-11.659) | | | 0.243 | 2.730 (0.580-12.858) | | 0.204 |  |
| M stage (M1 vs. M0) | 272 | 0.448 (0.125-1.604) | | | 0.217 | 0.425 (0.117-1.543) | | 0.194 |  |
| Pathologic stage (Stage III&IV vs. Stage I&Stage II) | 349 | 0.253 (0.054-1.188) | | | 0.082 | 0.238 (0.050-1.140) | | 0.073 |  |
| Vascular invasion (Yes vs. No) | 317 | 0.731 (0.486-1.099) | | | 0.132 | 0.725 (0.481-1.091) | | 0.123 |  |
| BIRC5/SKP2 (High expression vs. Low expression) | 373 | 0.593 (0.401-0.876) | | | **0.009** | 0.711 (0.480-1.053) | | 0.089 |  |
