# Supplementary material for: Development of a prognostic model for anoikis and identifies hub genes in hepatocellular carcinoma
Source: Sci Rep. 2023 Sep 7;13:14723. doi: 10.1038/s41598-023-41139-9 (PMC10484901; doi:10.1038/s41598-023-41139-9)
Supplement: Supplementary file 13 — Supplementary Table S6. [file 41598_2023_41139_MOESM13_ESM.docx]

**Supplementary Table S6**. Correlation of SKP2 expression levels with clinicopathological characteristics of HCC in the TMA cohort.

| **Characteristic** | **Low expression of SKP2** | **High expression of SKP2** | **p** |
| --- | --- | --- | --- |
| Gender |  |  | **0.002** |
| Male | 10(52.6%) | 59(85.5%) |  |
| Female | 9(47.4%) | 10(14.5%) |  |
| Age (year) |  |  | 0.741 |
| ≤60 | 11(57.9%) | 37(53.6%) |  |
| >60 | 8(42.1%) | 32 (46.4%) |  |
| Smoking history |  |  | 0.094 |
| No | 14(73.7%) | 36(52.2%) |  |
| Yes | 5(26.3%) | 33(47.8%) |  |
| Drinking history |  |  | **0.005** |
| No | 18(94.7%) | 42(60.9%) |  |
| Yes | 1(5.3%) | 27(39.1%) |  |
| Cirrhosis history |  |  | 0.634 |
| No | 6(31.6%) | 18(26.1%) |  |
| Yes | 13(68.4%) | 51(73.9%) |  |
| T Stage |  |  | 0.152 |
| T1&T2 | 18(94.7%) | 56(81.2%) |  |
| T3&T4 | 1(5.3%) | 13(18.8%) |  |
| N Stage |  |  | 0.453 |
| N0 | 19(100.0%) | 67(97.1%) |  |
| N1 | 0(0.0%) | 2(2.9%) |  |
| Pathologic stage |  |  | 0.152 |
| Stage Ⅰ&Ⅱ | 18(94.7%) | 56(81.2%) |  |
| Stage Ⅲ&Ⅳ | 1(5.3%) | 13(18.8%) |  |
| AFP |  |  | 0.304 |
| ≤6.7 IU/ml | 5(26.3%) | 27(39.1%) |  |
| >6.7 IU/ml | 14(73.7%) | 42(60.9%) |  |
| TB |  |  | 0.148 |
| ≤26μmol/L | 19(100.0%) | 62(89.9%) |  |
| >26μmol/L | 0(0.0%) | 7(10.1%) |  |
| ALB |  |  | 0.226 |
| <40 g/L | 2(10.5%) | 16(23.2%) |  |
| ≥40 g/L | 17(89.5%) | 53(76.8%) |  |
| Hepatits B virus inffection |  |  | 0.298 |
| No | 6(31.6%) | 14(20.3%) |  |
| Yes | 13(68.4%) | 55(79.7%) |  |
| HBV-DNA |  |  | 0.920 |
| ≤500IU/ml | 9(47.4%) | 33(47.8%) |  |
| >500IU/ml | 4(21.1%) | 17(24.6%) |  |
| Missing | 6(31.6%) | 19(27.5%) |  |
| Tumor size | 4.45±2.91 | 4.38±3.07 | 0.933 |
| Microvascular invasion |  |  | 0.905 |
| No | 8(42.1%) | 28(40.6%) |  |
| Yes | 11(57.9%) | 41(59.4%) |  |
| Satellite nodules |  |  | 0.844 |
| No | 15(78.9%) | 53(76.8%) |  |
| Yes | 4(21.1%) | 16(23.2%) |  |
| Liver capsule infiltration |  |  | 0.961 |
| No | 17(89.5%) | 62(89.9%) |  |
| Yes | 2(10.5%) | 7(10.1%) |  |
| Portal vein invasion |  |  | 0.283 |
| No | 19(100.0%) | 65(94.2%) |  |
| Yes | 0(0.0%) | 4(5.8%) |  |
| Portal vein embolus |  |  | 0.656 |
| No | 17(89.5%) | 59(85.5%) |  |
| Yes | 2(10.5%) | 10(14.5%) |  |
| Histologic grade |  |  | 0.255 |
| G1&G2 | 15(78.9%） | 45(65.2%) |  |
| G3&G4 | 4(21.1%) | 24(34.8%) |  |
