# Supplementary material for: Development of a prognostic model for anoikis and identifies hub genes in hepatocellular carcinoma
Source: Sci Rep. 2023 Sep 7;13:14723. doi: 10.1038/s41598-023-41139-9 (PMC10484901; doi:10.1038/s41598-023-41139-9)
Supplement: Supplementary file 14 — Supplementary Table S7. [file 41598_2023_41139_MOESM14_ESM.docx]

**Supplementary Table S7**. Univariate Cox regression analysis and multivariate regression analyses of PFI in the TMA cohort

| 1. **BIRC5 and SKP2 progression-free interval（PFI）univariate analysis** | | | | | | | | | |
| --- | --- | --- | --- | --- | --- | --- | --- | --- | --- |
| **Characteristics** | | **Total(N)** | | **Univariate analysis** | | | | | |
|  |  |  |  | **Hazard ratio (95% CI)** | | **P value** | | | |
| Gender(Female vs. Male) | | 88 | | 0.471(0.140-1.578) | | 0.222 | | | |
| Age (>60 vs. ≤60) | | 88 | | 1.396(0.613-3.060) | | 0.443 | | | |
| T Stage(T3&T4 vs. T1&T2) | | 88 | | 7.034(3.040-16.279) | | **<0.001** | | | |
| N Stage(N1 vs N0) | | 88 | | 2.615(0.349-19.586) | | 0.349 | | | |
| Pathologic stage(Stage Ⅲ&Ⅳ vs. StageⅠ&Ⅱ) | | 88 | | 7.034(3.040-16.279) | | **<0.001** | | | |
| Histologic grade(G3&G4 vs. G1&G2) | | 88 | | 2.732(1.225-6.090) | | **0.014** | | | |
| AFP(IU/ml)(>6.7 vs.≤6.7) | | 88 | | 1.914(0.760-4.825) | | 0.168 | | | |
| Vascular invasion(Yes vs. No) | | 88 | | 2.081(0.862-5.024) | | 0.103 | | | |
| BIRC5(High expression vs. Low expression ) | | 88 | | 8.290(1.118-61.468) | | **0.039** | | | |
| SKP2(High expression vs. Low expression ) | | 88 | | 8.226(1.109-61.000) | | **0.039** | | | |
| 1. **BIRC5 and SKP2 progression-free interval（PFI）multivariate analysis** | | | | | | | | |  |
| **Characteristics** | **Total(N)** | | **BIRC5 Multivariate analysis** | | | | **SKP2 Multivariate analysis** | |  |
|  |  |  | **Hazard ratio (95% CI)** | | **P value** | | **Hazard ratio (95% CI)** | **P value** |  |
| Pathologic stage(Stage Ⅲ&Ⅳ vs. StageⅠ&Ⅱ) | 88 | | 5.288(1.899-14.392) | | **0.001** | | 5.551(1.977-15.585) | **0.001** |  |
| Histologic grade(G3&G4 vs. G1&G2) | 88 | | 2.025(0.878-4.672) | | 0.098 | | 1.904(0.800-4.531) | 0.146 |  |
| AFP(IU/ml)(>6.7 vs.≤6.7) | 88 | | 0.997(0.370-2.686) | | 0.996 | | 1.468(0.550-3.917) | 0.444 |  |
| Vascular invasion(Yes vs. No) | 88 | | 0.974(0.340-2.795) | | 0.961 | | 0.903(0.305-2.679) | 0.855 |  |
| BIRC5/SKP2 (High expression vs. Low expression) | 88 | | 4.670(0.594-36.718) | | 0.143 | | 6.889(0.907-52.291) | 0.062 |  |
